# Impact of the metabolic syndrome on cardiopulmonary morbidity and mortality in individuals with lung function impairment: a prospective cohort study of the Danish general population

**DOI:** 10.1016/j.lanepe.2023.100759

**Published:** 2023-11-06

**Authors:** Jacob Louis Marott, Truls Sylvan Ingebrigtsen, Yunus Çolak, Hannu Kankaanranta, Per Sigvald Bakke, Jørgen Vestbo, Børge Grønne Nordestgaard, Peter Lange

**Affiliations:** a The Copenhagen City Heart Study, Copenhagen University Hospital – Bispebjerg and Frederiksberg, Copenhagen, Denmark; b The Copenhagen General Population Study, Copenhagen University Hospital – Herlev and Gentofte, Herlev, Denmark; c Department of Internal Medicine, Respiratory Section, Copenhagen University Hospital – Herlev and Gentofte, Herlev, Denmark; d Krefting Research Centre, Department of Internal Medicine and Clinical Nutrition, Institute of Medicine, Sahlgrenska Academy, University of Gothenburg, Gothenburg, Sweden; e Department of Respiratory Medicine, Seinäjoki Central Hospital, Seinäjoki, Finland; f Faculty of Medicine and Health Technology, Tampere University, Tampere, Finland; g Department of Clinical Science, University of Bergen, Norway; h Division of Infection, Immunity and Respiratory Medicine, University of Manchester, Manchester, United Kingdom; i North West Lung Centre, Manchester University NHS Foundation Trust, Manchester, United Kingdom; j Department of Clinical Biochemistry, Copenhagen University Hospital – Herlev and Gentofte, Herlev, Denmark; k Department of Clinical Medicine, Faculty of Health and Medical Sciences, University of Copenhagen, Copenhagen, Denmark; l Department of Public Health, Section of Epidemiology, University of Copenhagen, Copenhagen, Denmark

**Keywords:** Preserved ratio impaired spirometry, PRISm, Airflow limitation, Cardiopulmonary morbidity, Mortality

## Abstract

**Background:**

Whether the metabolic syndrome plays a role for the prognosis of individuals with lung function impairment (preserved ratio impaired spirometry (PRISm) or airflow limitation) is unclear. We hypothesised that the metabolic syndrome in individuals with lung function impairment is associated with increased cardiopulmonary morbidity and mortality.

**Methods:**

The Copenhagen General Population Study was initiated in 2003 based on a random sample of white men and women aged 20–100 years drawn from the Danish general population. The risk of ischemic heart disease/heart failure, respiratory disease, and all-cause mortality was analysed with Cox models adjusted for age, sex, current smoking, and asthma during 15 years of follow-up.

**Findings:**

Among 106,845 adults, 86,159 had normal lung function, 6126 had PRISm, and 14,560 had airflow limitation. We observed 10,448 hospital admissions for ischemic heart disease/heart failure, 21,140 for respiratory disease, and 11,125 deaths. Individuals with versus individuals without the metabolic syndrome generally had higher 5-year absolute risk of all outcomes, including within those with normal lung function, mild-moderate-severe PRISm, and very mild-mild-moderate-severe airflow limitation alike. Compared to individuals without the metabolic syndrome and with normal lung function, those with both the metabolic syndrome and severe PRISm had hazard ratios of 3.74 (95% CI: 2.53–5.55; p < 0.0001) for ischemic heart disease/heart failure, 5.02 (3.85–6.55; p < 0.0001) for respiratory disease, and 5.32 (3.76–7.54; p < 0.0001) for all-cause mortality. Corresponding hazard ratios in those with both the metabolic syndrome and severe airflow limitation were 2.89 (2.34–3.58; p < 0.0001) for ischemic heart disease/heart failure, 5.98 (5.28–6.78; p < 0.0001) for respiratory disease, and 4.16 (3.50–4.95; p < 0.0001) for all-cause mortality, respectively. The metabolic syndrome explained 13% and 27% of the influence of PRISm or airflow limitation on ischemic heart disease/heart failure and all-cause mortality.

**Interpretation:**

The metabolic syndrome conferred increased risk of cardiopulmonary morbidity and mortality at all levels of lung function impairment.

**Funding:**

Danish Lung Foundation, 10.13039/100007405Danish Heart Foundation, Capital Region of Copenhagen, and Boehringer Ingelheim. JV is supported by the 10.13039/100014653NIHR Manchester BRC.

**Research in context**

**Evidence before this study**

We searched PubMed for available human articles in English until October 2, 2023, using the search phrases “preserved ratio impaired spirometry”, “PRISm”, “lung function impairment”, “restrictive spirometry”, “airflow limitation”, “metabolic syndrome”, and “systemic inflammation”, separately and in different combinations. A recent study identified smoking, asthma, and, in particular, overweight as important predictors of preserved ratio impaired spirometry (PRISm). The metabolic syndrome is globally on the rise, and it is associated with obesity, low lung function, and a high risk of cardiovascular disease. The role of low-grade systemic inflammation in PRISm and airflow limitation and their association with the metabolic syndrome is still debated.

**Added value of this study**

This study focused on the role of the metabolic syndrome in cardiopulmonary morbidity and mortality in individuals with PRISm and airflow limitation. Individuals with versus individuals without the metabolic syndrome generally had higher 5-year absolute risk of ischemic heart disease/heart failure, respiratory disease, and deaths, including within those with normal lung function, mild-moderate-severe PRISm, and very mild-mild-moderate-severe airflow limitation alike. The metabolic syndrome explained 13% and 27% of the influence of PRISm or airflow limitation on ischemic heart disease/heart failure and all-cause mortality.

**Implications of all the available evidence**

We have shown that the combination of the metabolic syndrome and poor lung function leads to a very high cardiopulmonary morbidity and mortality risk. If successful, improvements in lung function and metabolic syndrome status could have a huge impact on public health. Interventions with a focus on physical activity, a healthy diet, and the implementation of specific therapies like Glucagon-like peptide-1 analogues to loose weight in high-risk individuals may reduce cardiopulmonary morbidity and mortality and improve both lung function and prognosis.

## Introduction

The association between impaired lung function and poor survival has repeatedly been shown in individuals with airflow limitation and in those with lung function impairment but without airflow limitation, characterised by reduction of both forced expiratory volume in 1 s (FEV1) and forced vital capacity (FVC), recently labelled as preserved ratio impaired spirometry (PRISm).^1^, ^2^, ^3^, ^4^ This association has been observed in general population cohorts and in cohorts comprising patients with clinical lung disease irrespective of tobacco smoking, and possible mechanisms have been discussed throughout the years.^5^, ^6^, ^7^, ^8^

In countries with a high sociodemographic index, as defined in the Global Burden of Disease Study,^9^ the two dominant risk factors for airflow limitation are active smoking and asthma, whereas in countries with a low sociodemographic index, indoor and outdoor air pollution and previous tuberculosis also contribute heavily.^10^ Risk factors for PRISm are not as well-described as for airflow limitation, but in 2022, a study based on the UK Biobank identified smoking, asthma, and, in particular, overweight and obesity as important predictors of PRISm.^11^

In recent decades, low-grade systemic inflammation has been suggested to play a role in the development of several chronic diseases. However, its importance for the development, manifestation, and prognosis of both airflow limitation and PRISm remains controversial.^12^^,^^13^ In this context, the role of the metabolic syndrome is relevant to explore, as this condition is globally on the rise, is associated with low-grade systemic inflammation, obesity, and low lung function, and is strongly related to cardiovascular disease–a prominent cause of morbidity and mortality in both airflow limitation and PRISm.^4^^,^^14^, ^15^, ^16^, ^17^

In the present study, we hypothesised that the metabolic syndrome in individuals with lung function impairment is associated with increased cardiopulmonary morbidity and mortality. We also assessed whether the associations between lung function impairment and these outcomes were mediated partly through the metabolic syndrome. For this purpose, we used data from the Copenhagen General Population Study, an ongoing contemporary study of more than 100,000 clinically well-characterised individuals from the Danish general population followed for up to 15 years with regards to cardiopulmonary morbidity and mortality.

## Methods

### Study design and population

The Copenhagen General Population Study is a contemporary prospective cohort study based on a random sample of the Danish general population, with ongoing enrolment since 2003. The sample was created using the Danish Central Person Registration number, which uniquely identifies all individuals living in Denmark. In total, 256,761 white men and women of Danish descent aged 20–100 years and living in the greater Copenhagen area were invited, and 43% participated. From these 109,328 individuals, we excluded those without spirometry (n = 1044) and those with missing values in one or more of the variables used to define the metabolic syndrome (n = 1439), leaving 106,845 individuals for analyses. All participants provided written informed consent and completed a detailed questionnaire, including information on symptoms and diseases (such as asthma), socio-economic status, physical activity, smoking habits, and medication use. A physical examination was conducted, followed by the collection of blood samples for biochemical testing. All available data besides the clinical outcomes during follow-up were collected on the day of the examination. The study was approved by an institutional review board and a Danish ethics committee (approval number: H-KF-01-144/01), and was conducted according to the Declaration of Helsinki.

### Spirometry and definition of lung function phenotypes

Lung function was determined during the physical examination with measurements of pre-bronchodilator FEV_1_ and FVC by trained healthcare professionals.^18^

The lower limit of normal (LLN) was defined as the 5th percentile, as recommended by the European Respiratory Society and the American Thoracic Society.^19^ For this, Z-scores are often used, but when it comes to the FEV_1_/FVC ratio, predicting the 5th percentile from a regression model should be preferred, as no assumption about normality is required. LLN was calculated from sex-specific 5% quantile reference equations for FEV_1_/FVC with adjustments for age squared and height.^18^ The reference equations were derived from the same ethnic group as the present study.

Z-scores for FEV_1_ were used to define the LLN for FEV_1_ and the severity of lung function impairment. A Z-score of −1.645 for FEV_1_ defined the cut-point for LLN.

1) Normal lung function was defined as FEV_1_/FVC ≥ LLN and a FEV_1_ Z-score ≥−1.645
2) PRISm was defined as FEV_1_/FVC ≥ LLN and a FEV_1_ Z-score <−1.645
3) Airflow limitation was defined as FEV_1_/FVC < LLN.

All individuals were hereby assigned to one of these three mutually exclusive clinical groups, referred to as the lung function phenotypes. We will refer to airflow limitation and PRISm in combination as the lung function impairment phenotype.

The PRISm and airflow limitation groups were also sub-categorised according to the severity of the lung function impairment by FEV_1_ Z-scores.

1) Mild: FEV_1_ Z-score ≥−2.5 and FEV_1_ Z-score <−1.645.
2) Moderate: FEV_1_ Z-score ≥−3.3 and FEV_1_ Z-score <−2.5.
3) Severe: FEV_1_ Z-score <−3.3.

Very mild lung function impairment was defined as a FEV_1_ Z-score ≥−1.645 within the airflow limitation group. The European Respiratory Society and the American Thoracic Society suggest a cut-point for the FEV_1_ Z-score of −4 between moderate and severe lung function impairment,^19^ but cut-points like −2.55 and −3 have previously been suggested.^20^^,^^21^ We chose a cut-point of −3.3 for the FEV_1_ Z-score (i.e., the 0.05 percentile), so that just 1 in every 2000 healthy individuals was expected to have a lower value of FEV_1_. In Supplementary Figure S1, the lung function phenotype classification according to cut-points of FEV_1_/FVC and FEV_1_ Z-scores is depicted.

### Laboratory measurements

We used non-fasting samples to measure plasma triglycerides, high-density lipoprotein (HDL) cholesterol, high-sensitivity C-reactive protein (HS-CRP), and fibrinogen using standard hospital assays.

### Definition of the metabolic syndrome

The metabolic syndrome was defined as fulfilling at least three of the following five criteria: 1) elevated plasma triglycerides (non-fasting triglycerides ≥1.7 mmol/L), 2) low HDL cholesterol (non-fasting HDL cholesterol <1.30 mmol/L in women and <1.03 mmol/L in men), 3) elevated blood glucose (non-fasting blood glucose >7.8 mmol/L and/or treatment with antidiabetic drugs), 4) hypertension (systolic blood pressure ≥130 mmHg or diastolic blood pressure ≥85 mmHg or treatment with antihypertensive drugs), and 5) central obesity (waist circumference ≥88 cm in women and ≥102 cm in men) in accordance with the recommendations from the American Heart Association.^22^^,^^23^ We adjusted the cut-point for blood glucose to accommodate for non-fasting values. The recommended cut-points for fasting plasma triglycerides and HDL cholesterol were used, as these lipids are much less affected by the use of non-fasting values compared with plasma glucose.^23^^,^^24^

### Clinical outcomes

The national Danish Civil Registration System provided information about all-cause mortality, including date of death and emigration, until the end of follow-up on December 13, 2018. Cause-specific mortality was obtained from the national Danish Causes of Death Registry, with the end of follow-up on December 31, 2016. Morbidity outcomes were based on hospitalisation discharge diagnoses obtained from the national Danish Patient Registry, with the end of follow-up on December 7, 2018.

Three outcomes of interest were considered primary outcomes.

1) All-cause mortality.
2) Ischemic heart disease (International Classification of Diseases (ICD)-10: I20–I25) and/or heart failure (ICD-10: I50) morbidity.
3) Respiratory disease (ICD-10: J00–J99) morbidity.

Two cause-specific mortality outcomes were considered secondary outcomes.

1) Cardiac (ICD-10: I00–I99) mortality.
2) Respiratory disease (ICD-10: J00–J99) mortality.

Individuals who emigrated were censored at the date of emigration, and none were lost to follow-up by using the unique Danish Central Person Registration number.

### Statistical analysis

For each of the three lung function phenotypes, when comparing individuals with and without the metabolic syndrome, Pearson's χ^2^ test was used for categorical variables, and for continuous variables, Student's t-test was applied when comparing means and the Kruskal–Wallis test when comparing medians (Table 1). Censoring was defined as the date of death, the date of emigration, or the end of follow-up, whichever came first. Death was chosen as a competing risk for morbidity outcomes, and death from causes other than the specific cause under consideration for cause-specific mortality outcomes. For each of the cardiovascular and respiratory outcomes, the cumulative incidence was estimated as 1 minus the Aalen-Johansen estimator. For all-cause mortality, the survival function was estimated by the Kaplan–Meier estimator. The median follow-up time was estimated by the reverse Kaplan–Meier method by reversing the event indicator so that the outcome of interest becomes being censored.^25^

**Table 1:** Baseline characteristics according to lung function impairment and the metabolic syndrome in 106,845 individuals in the Copenhagen General Population Study.

	Normal lung function (n = 86,159)			PRISm (n = 6126)			Airflow limitation (n = 14,560)		
	Without MetS (n = 66,847)	With MetS (n = 19,312)	p-value	Without MetS (n = 3726)	With MetS (n = 2400)	p-value	Without MetS (n = 11,263)	With MetS (n = 3297)	p-value
*General characteristics*									
Male sex—no. (%)	28,180 (42)	10,162 (53)	<0.0001	1407 (38)	1234 (51)	<0.0001	5208 (46)	1881 (57)	<0.0001
Age—years	57 ± 13	60 ± 12	<0.0001	57 ± 12	60 ± 11	<0.0001	61 ± 13	63 ± 12	<0.0001
FEV_1_									
Mean—L	3.2 ± 0.8	3.0 ± 0.8	<0.0001	2.2 ± 0.6	2.2 ± 0.6	0.91	2.4 ± 0.8	2.3 ± 0.8	<0.0001
Percent of predicted value	101 ± 13	97 ± 12	<0.0001	71 ± 8	69 ± 8	<0.0001	81 ± 19	74 ± 19	<0.0001
Z-score	0.1 ± 0.9	−0.2 ± 0.9	<0.0001	−2.1 ± 0.5	−2.2 ± 0.5	<0.0001	−1.3 ± 1.3	−1.8 ± 1.3	<0.0001
FVC									
Mean—L	4.0 ± 1.0	3.9 ± 1.0	<0.0001	2.9 ± 0.8	2.9 ± 0.8	0.59	3.9 ± 1.2	3.6 ± 1.2	<0.0001
Percent of predicted value	102 ± 13	97 ± 13	<0.0001	74 ± 10	72 ± 9	<0.0001	101 ± 20	93 ± 21	<0.0001
Z-score	0.2 ± 0.9	−0.2 ± 0.9	<0.0001	−1.9 ± 0.6	−2.0 ± 0.6	<0.0001	0.1 ± 1.4	−0.5 ± 1.4	<0.0001
FEV_1_/FVC	0.79 ± 0.05	0.79 ± 0.05	<0.0001	0.76 ± 0.06	0.77 ± 0.06	0.059	0.63 ± 0.07	0.62 ± 0.07	<0.0001
*Symptoms*									
Dyspnoea (mMRC ≥2)—no. (%)	2851 (4)	2421 (13)	<0.0001	573 (15)	699 (29)	<0.0001	1453 (13)	807 (25)	<0.0001
Chronic mucus hypersecretion—no. (%)	4014 (6)	1915 (10)	<0.0001	497 (13)	415 (17)	<0.0001	1840 (16)	703 (21)	<0.0001
Frequent exacerbations (>5/10 years)—no. (%)	734 (1)	374 (2)	<0.0001	150 (4)	122 (5)	0.052	490 (4)	215 (7)	<0.0001
Exposure to dust and fumes—no. (%)	5219 (8)	2465 (13)	<0.0001	455 (12)	497 (21)	<0.0001	1407 (13)	631 (19)	<0.0001
Wheezing—no. (%)	7476 (11)	3821 (20)	<0.0001	1079 (29)	993 (42)	<0.0001	3253 (29)	1373 (42)	<0.0001
*Lifestyle factors*									
Body mass index									
Mean—kg/m^2^	25 ± 3	30 ± 4	<0.0001	26 ± 4	31 ± 5	<0.0001	25 ± 3	29 ± 4	<0.0001
≥25—no. (%)	30,813 (46)	17,437 (90)	<0.0001	1914 (51)	2210 (92)	<0.0001	4548 (40)	2814 (85)	<0.0001
≥30—no. (%)	5137 (8)	8290 (43)	<0.0001	519 (14)	1280 (53)	<0.0001	721 (6)	1209 (37)	<0.0001
Waist circumference—cm	86 ± 11	101 ± 11	<0.0001	89 ± 13	106 ± 13	<0.0001	87 ± 11	102 ± 12	<0.0001
Smoking history			<0.0001			<0.0001			<0.0001
Never-smoker—no. (%)	29,297 (46)	7273 (39)		1178 (33)	620 (27)		2851 (26)	578 (18)	
Former smoker—no. (%)	25,951 (41)	8162 (44)		1349 (38)	924 (40)		4700 (43)	1567 (49)	
Current smoker—no. (%)	8615 (13)	3120 (17)		1057 (29)	764 (33)		3342 (31)	1052 (33)	
Consumption in pack-years, median [IQR]	12 [5–24]	19 [8–32]	<0.0001	23 [10–36]	30 [15–45]	<0.0001	24 [11–39]	30 [18–46]	<0.0001
Alcohol intake—no. (%)			<0.0001			<0.0001			<0.0001
Never	4371 (7)	1908 (10)		330 (10)	296 (14)		916 (8)	371 (12)	
Moderate	48,922 (76)	13,051 (72)		2445 (71)	1438 (66)		7587 (70)	2055 (66)	
High	11,040 (17)	3264 (18)		676 (20)	457 (21)		2304 (21)	699 (22)	
Physical activity—no. (%)			<0.0001			<0.0001			<0.0001
Low	2835 (4)	1790 (9)		348 (9)	391 (17)		761 (7)	454 (14)	
Moderate	25,680 (39)	9534 (50)		1832 (50)	1265 (53)		4732 (42)	1588 (49)	
High	37,895 (57)	7804 (41)		1494 (41)	711 (30)		5665 (51)	1215 (37)	
Education—no. (%)			<0.0001			<0.0001			<0.0001
<Middle school	4937 (7)	2588 (13)		430 (12)	421 (18)		1397 (12)	590 (18)	
Middle school	23,927 (36)	8677 (45)		1691 (46)	1232 (52)		4594 (41)	1554 (47)	
High school	24,486 (37)	5603 (29)		1151 (31)	567 (24)		3465 (31)	786 (24)	
University	13,304 (20)	2378 (12)		438 (12)	168 (7)		1754 (16)	350 (11)	
*Comorbidities and clinical measurements*									
Current asthma—no. (%)	2749 (4)	927 (5)	<0.0001	333 (9)	247 (10)	0.091	1576 (14)	509 (16)	0.040
Asthma as child—no. (%)	9307 (14)	2269 (12)	<0.0001	529 (14)	308 (13)	0.13	1841 (16)	460 (14)	0.001
Diabetes—no. (%)	1008 (2)	1984 (10)	<0.0001	95 (3)	404 (17)	<0.0001	225 (2)	418 (13)	<0.0001
Blood pressure—mmHg									
Systolic	139 ± 21	149 ± 19	<0.0001	139 ± 22	149 ± 21	<0.0001	141 ± 22	149 ± 20	<0.0001
Diastolic	83 ± 11	88 ± 11	<0.0001	83 ± 12	88 ± 12	<0.0001	83 ± 11	87 ± 12	<0.0001
Blood pressure medication—no. (%)	9101 (15)	5772 (33)	<0.0001	677 (19)	839 (37)	<0.0001	1973 (20)	1078 (37)	<0.0001
*Blood samples*									
Glucose >7.8 mmol/L—no. (%)	853 (1)	1637 (8)	<0.0001	49 (1)	288 (12)	<0.0001	154 (1)	311 (9)	<0.0001
HDL cholesterol <1.3 (men)/1.03 (women) mmol/L—no. (%)	4475 (7)	10,912 (57)	<0.0001	278 (7)	1319 (55)	<0.0001	763 (7)	1764 (54)	<0.0001
Triglycerides ≥1.7 mmol/L—no. (%)	12,884 (19)	17,288 (90)	<0.0001	821 (22)	2132 (89)	<0.0001	2291 (20)	2910 (88)	<0.0001
Fibrinogen ≥14 μmol/L—no. (%)	5741 (9)	2982 (15)	<0.0001	757 (20)	675 (28)	<0.0001	1473 (13)	658 (20)	<0.0001
HS-CRP ≥3 mg/L—no. (%)	7851 (12)	5083 (27)	<0.0001	957 (26)	1137 (48)	<0.0001	1987 (18)	1100 (34)	<0.0001
IgE ≥150 IU/mL—no. (%)	2436 (8)	891 (10)	<0.0001	141 (11)	126 (14)	0.016	802 (14)	262 (16)	0.072
Eosinophils ≥500 cells/μL—no. (%)	1718 (3)	665 (3)	<0.0001	121 (3)	107 (4)	0.018	467 (4)	178 (5)	0.002

Data are n (%), mean ± SD, or median [IQR]. Abbreviations: PRISm, preserved ratio impaired spirometry; MetS, metabolic syndrome; FEV_1_, forced expiratory volume in 1 s; FVC, forced vital capacity; mMRC, modified Medical Research Council dyspnoea scale; IQR, interquartile range; HDL, high-density lipoprotein; HS-CRP, high-sensitivity C-reactive protein; IgE, immunoglobulin E.

The association between lung function phenotype and all-cause mortality was analysed with Cox proportional hazards regression analysis using follow-up time as timescale. For all other outcomes, competing risk analyses were performed with cause-specific Cox regression models using follow-up time as timescale. The airflow limitation and PRISm groups were also sub-categorised into stages of lung function impairment based on FEV_1_ Z-scores: very mild (only airflow limitation), mild, moderate, and severe. Lung function, in the form of FEV_1_ Z-scores, was analysed both as a continuous variable with restricted cubic splines and as a categorical variable defined by FEV_1_ Z-score stages. Interactions were tested multiplicatively in Cox models and additively in Aalen's additive hazards models.

Mediation analyses were performed for all primary outcomes with lung function (both lung function impairment phenotype and FEV_1_ Z-score) as exposure and the metabolic syndrome as mediator, and with the metabolic syndrome as exposure and lung function (FEV_1_ Z-score) as mediator. The mediation analyses were performed to estimate the proportions mediated, and the 95% confidence intervals were based on 10,000 bootstrap samples. HS-CRP (log-transformed) and fibrinogen were included in Cox models to analyse the associations between systemic inflammation and outcomes and in mediation analyses with the biomarkers as both exposures and mediators.

All regression models were adjusted for age, sex, current smoking, and asthma. The key assumption of the Cox regression, the proportionality assumption, was tested with the score process test. Misspecifications of the functional forms of age and FEV_1_ Z-score in the Cox models were tested by plotting these continuous covariates against the cumulative sums of martingale-based residuals and comparing them to random realisations under the models.^26^ All analyses were performed with R version 4.2.2 (R Foundation for Statistical Computing, Vienna, Austria), and a two-sided p-value < 0.05 was chosen to indicate statistical significance.

### Role of funding sources

The study was supported by The Danish Lung Foundation, The Danish Heart Foundation, The Capital Region of Copenhagen, and Boehringer Ingelheim. JV is supported by the National Institute for Health and Care Research (NIHR) Manchester Biomedical Research Centre (BRC). The funding sources provided unrestricted grants and did not take any part in the design or conduct of the study; collection, analysis, or interpretation of the data; preparation, review, or approval of the manuscript; or decision to submit the manuscript for publication.

## Results

### Lung function phenotypes and the metabolic syndrome

Out of 106,845 individuals, 86,159 (81%) had normal lung function, 6126 (6%) had PRISm, and 14,560 (14%) had airflow limitation. The metabolic syndrome was observed in 39% with PRISm and in 23% with airflow limitation, compared to 22% with normal lung function (Table 1). In all three lung function phenotypes, the metabolic syndrome was more prevalent among ever-smokers than never-smokers; however, it was higher in never-smokers with PRISm than in ever-smokers with airflow limitation or normal lung function (Supplementary Figure S2).

As expected, across the three lung function phenotypes, the metabolic syndrome was associated with the variables used to define the condition: blood glucose, diabetes mellitus, plasma triglycerides, HDL cholesterol, blood pressure and blood pressure medication, and with waist circumference (Table 1). Furthermore, within each lung function phenotype, the metabolic syndrome was associated with male sex, dyspnea, chronic mucus hypersecretion, previous exposure to dust and fumes, wheezing, a high body mass index, high tobacco consumption, a low educational level, physical inactivity, and high levels of fibrinogen and HS-CRP (Table 1). Baseline characteristics according to severity of lung function impairment and the metabolic syndrome are presented for individuals with PRISm (Supplementary Table S1) and airflow limitation (Supplementary Table S2).

### The metabolic syndrome and outcomes within lung function phenotypes

First, the association of the metabolic syndrome with risk of morbidity and mortality was investigated within the different lung function phenotypes.

During a median follow-up time of 9.9 years (interquartile range: 7.2–12.3), we recorded 10,448 hospital admissions for ischemic heart disease/heart failure and 21,140 for respiratory disease. Among the 11,125 deaths observed, 3304 were cardiac deaths and 2556 were respiratory disease deaths.

**Fig. 1:**
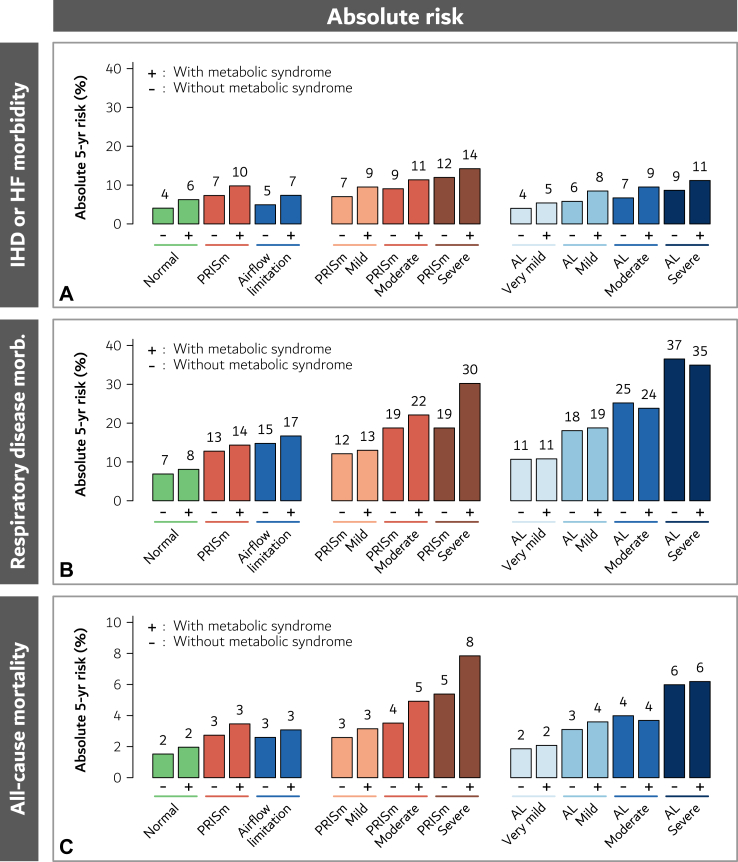
Absolute risk of ischemic heart disease or heart failure morbidity (Panel A), respiratory disease morbidity (Panel B), and all-cause mortality (Panel C) according to lung function and the metabolic syndrome with adjustment for age, sex, asthma, and smoking. Abbreviations: Normal, normal lung function; PRISm, preserved ratio impaired spirometry; AL, airflow limitation; IHD, ischemic heart disease; HF, heart failure. The severity of the lung function impairment was categorised in very mild (only AL), mild, moderate, and severe, based on FEV_1_ Z-scores.

Individuals with versus individuals without the metabolic syndrome generally had higher 5-year absolute risk of all outcomes, including within those with normal lung function, mild-moderate-severe PRISm, and very mild-mild-moderate-severe airflow limitation alike (Fig. 1).

**Fig. 2:**
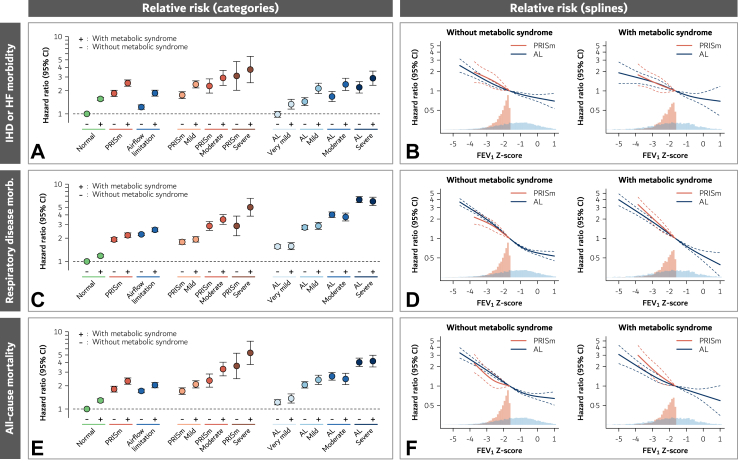
Risk of ischemic heart disease or heart failure morbidity (Panels A and B), respiratory disease morbidity (Panels C and D), and all-cause mortality (Panels E and F) according to lung function in categories (Panels A, C, and E) and continuously (Panels B, D, and F) and the metabolic syndrome with adjustment for age, sex, asthma, and smoking. Abbreviations: Normal, normal lung function; PRISm, preserved ratio impaired spirometry; AL, airflow limitation; IHD, ischemic heart disease; HF, heart failure; CI, confidence interval. The severity of the lung function impairment was categorised in very mild (only AL), mild, moderate, and severe, based on FEV_1_ Z-scores.

Compared to individuals without the metabolic syndrome and with normal lung function, those with both the metabolic syndrome and severe PRISm had hazard ratios of 3.74 (95% CI: 2.53–5.55; p < 0.0001) for ischemic heart disease/heart failure, 5.02 (3.85–6.55; p < 0.0001) for respiratory disease, and 5.32 (3.76–7.54; p < 0.0001) for all-cause mortality (Table 2, Fig. 2 (left part), and Supplementary Figures S3–S5). Corresponding hazard ratios in those with both the metabolic syndrome and severe airflow limitation were 2.89 (2.34–3.58; p < 0.0001) for ischemic heart disease/heart failure, 5.98 (5.28–6.78; p < 0.0001) for respiratory disease, and 4.16 (3.50–4.95; p < 0.0001) for all-cause mortality, respectively.

**Table 2:** Risk of ischemic heart disease or heart failure morbidity, respiratory disease morbidity, and all-cause mortality according to lung function phenotype and the metabolic syndrome with adjustment for age, sex, asthma, and smoking in 106,845 individuals from the Copenhagen General Population Study.

	Metabolic syndrome	No. of individuals	IHD or heart failure morbidity		Respiratory disease morbidity		All-cause mortality	
			Hazard ratio (95% CI), p-value	p-value^a^	Hazard ratio (95% CI), p-value	p-value^a^	Hazard ratio (95% CI), p-value	p-value^a^
**Overall**		**106,845**						
Normal (reference)	**−**	66,847	1.00 (reference)	<0.0001	1.00 (reference)	<0.0001	1.00 (reference)	<0.0001
Normal	**+**	19,312	1.56 (1.49–1.64), p < 0.0001		1.18 (1.14–1.23), p < 0.0001		1.29 (1.23–1.36), p < 0.0001	
PRISm	**−**	3726	1.84 (1.68–2.02), p < 0.0001	<0.0001	1.92 (1.81–2.04), p < 0.0001	0.005	1.81 (1.66–1.97), p < 0.0001	0.0001
PRISm	**+**	2400	2.50 (2.28–2.75), p < 0.0001		2.18 (2.03–2.34), p < 0.0001		2.30 (2.09–2.52), p < 0.0001	
Airflow limitation	**−**	11,263	1.22 (1.15–1.31), p < 0.0001	<0.0001	2.24 (2.15–2.33), p < 0.0001	<0.0001	1.71 (1.62–1.81), p < 0.0001	<0.0001
Airflow limitation	**+**	3297	1.85 (1.70–2.02), p < 0.0001		2.56 (2.42–2.72), p < 0.0001		2.04 (1.88–2.21), p < 0.0001	
**PRISm groups**		**6126**						
PRISm Mild	**−**	3100	1.75 (1.58–1.94), p < 0.0001	<0.0001	1.78 (1.67–1.91), p < 0.0001	0.13	1.70 (1.55–1.88), p < 0.0001	0.005
PRISm Mild	**+**	1919	2.41 (2.16–2.67), p < 0.0001		1.93 (1.78–2.09), p < 0.0001		2.08 (1.87–2.32), p < 0.0001	
PRISm Moderate	**−**	525	2.29 (1.86–2.83), p < 0.0001	0.12	2.88 (2.52–3.29), p < 0.0001	0.05	2.33 (1.92–2.84), p < 0.0001	0.02
PRISm Moderate	**+**	384	2.92 (2.34–3.65), p < 0.0001		3.49 (3.01–4.04), p < 0.0001		3.29 (2.68–4.03), P < 0.0001	
PRISm Severe	**−**	101	3.10 (2.02–4.75), p < 0.0001	0.52	2.88 (2.15–3.86), p < 0.0001	0.006	3.61 (2.47–5.27), p < 0.0001	0.14
PRISm Severe	**+**	97	3.74 (2.53–5.55), p < 0.0001		5.02 (3.85–6.55), p < 0.0001		5.32 (3.76–7.54), p < 0.0001	
**Airflow limitation groups**		**14,560**						
AL Very mild	**−**	7112	0.98 (0.90–1.07), p = 0.71	0.0003	1.56 (1.48–1.65), p < 0.0001	0.86	1.22 (1.13–1.32), p < 0.0001	0.14
AL Very mild	**+**	1582	1.33 (1.15–1.54), p < 0.0001		1.58 (1.42–1.75), p < 0.0001		1.37 (1.20–1.57), p < 0.0001	
AL Mild	**−**	2295	1.44 (1.28–1.62), p < 0.0001	<0.0001	2.75 (2.57–2.94), P < 0.0001	0.45	2.05 (1.87–2.26), p < 0.0001	0.07
AL Mild	**+**	846	2.14 (1.83–2.49), p < 0.0001		2.88 (2.59–3.20), p < 0.0001		2.38 (2.08–2.74), p < 0.0001	
AL Moderate	**−**	1115	1.68 (1.44–1.95), p < 0.0001	0.002	4.02 (3.71–4.37), p < 0.0001	0.33	2.65 (2.36–2.97), p < 0.0001	0.43
AL Moderate	**+**	513	2.41 (2.01–2.89), p < 0.0001		3.76 (3.34–4.23), p < 0.0001		2.45 (2.07–2.90), p < 0.0001	
AL Severe	**−**	741	2.20 (1.86–2.61), p < 0.0001	0.04	6.30 (5.77–6.89), p < 0.0001	0.49	4.02 (3.55–4.56), p < 0.0001	0.74
AL Severe	**+**	356	2.89 (2.34–3.58), p < 0.0001		5.98 (5.28–6.78), p < 0.0001		4.16 (3.50–4.95), p < 0.0001	

Abbreviations: PRISm, preserved ratio impaired spirometry; AL, airflow limitation; IHD, ischemic heart disease; CI, confidence interval.

a Within each lung function group, individuals with and without the metabolic syndrome are compared.

The increased risk for all-cause mortality was driven both by increased cardiac mortality and increased respiratory disease mortality (Supplementary Figures S6–S7).

### Moderation analyses

Second, for each morbidity and mortality outcome, we assessed whether or not the increased risk of outcomes conferred by the metabolic syndrome was of similar size in PRISm and airflow limitation.

There was no evidence of additive or multiplicative interaction between the metabolic syndrome and lung function phenotypes on outcome, indicating that the metabolic syndrome was equally important in PRISm and airflow limitation, except for respiratory disease morbidity in the additive model, where the metabolic syndrome was associated with a slightly higher risk in airflow limitation compared to PRISm (Supplementary Figure S8).

### Mediation analyses

Third, the proportion of the influence of lung function on morbidity and mortality explained by the metabolic syndrome and systemic inflammation was estimated.

**Fig. 3:**
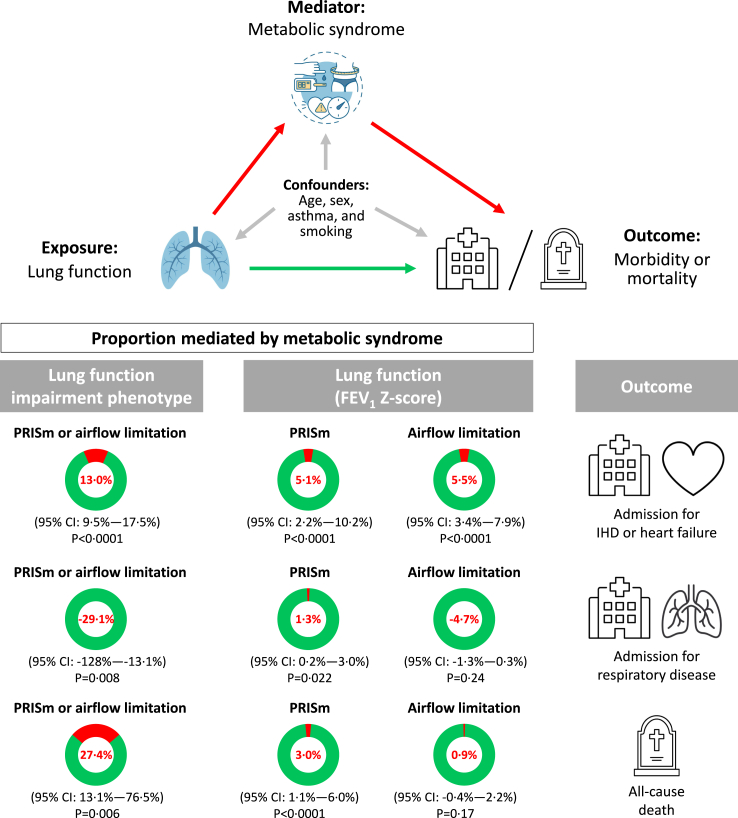
Mediation analysis between the exposure lung function (both lung function impairment phenotype and FEV_1_ Z-score), the mediator the metabolic syndrome, and the outcomes ischemic heart disease or heart failure morbidity, respiratory disease morbidity, and all-cause mortality. Abbreviations: PRISm, preserved ratio impaired spirometry; IHD, ischemic heart disease; CI, confidence interval.

The metabolic syndrome explained 13% (95% CI: 9%–18%; p < 0.0001) of the influence of lung function impairment phenotype (PRISm or airflow limitation) on ischemic heart disease/heart failure and 27% (13%–76%; p = 0.006) on all-cause mortality (Fig. 3). In those with PRISm alone based on FEV_1_ Z-score, the metabolic syndrome only explained 5% (2%–10%; p < 0.0001) of the influence of lung function on ischemic heart disease/heart failure and 3% (1%–6%; p < 0.0001) on all-cause mortality. Correspondingly, in those with airflow limitation alone based on FEV_1_ Z-score, 5% (3%–8%; p < 0.0001) was explained for ischemic heart disease/heart failure and 1% (0%–2%; p = 0.17) for all-cause mortality. No mediation was present for respiratory disease (Fig. 3).

**Fig. 4:**
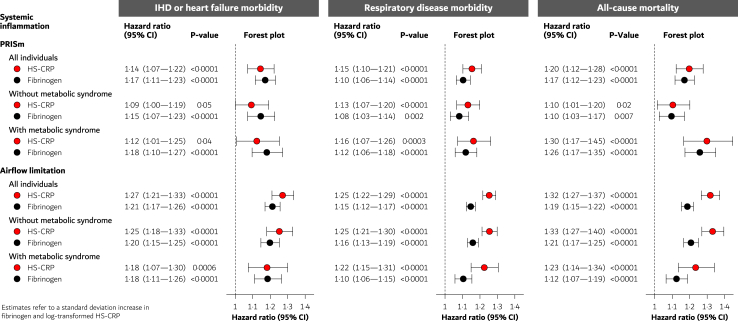
Low-grade systemic inflammation and risk of ischemic heart disease or heart failure morbidity, respiratory disease morbidity, and all-cause mortality in all individuals with PRISm or airflow limitation, or in those with or without the metabolic syndrome. All models were adjusted for age, sex, asthma, and smoking. Abbreviations: PRISm, preserved ratio impaired spirometry; HS-CRP, high-sensitivity C-reactive protein; IHD, ischemic heart disease; CI, confidence interval.

Higher values of HS-CRP and fibrinogen were associated with higher risks of all outcomes (Fig. 4), and the explained proportions related to these biomarkers were slightly higher than those observed for the metabolic syndrome (Supplementary Figures S9–S10).

### Lung function on risk of outcomes by metabolic syndrome status

Lower lung function was associated with a higher risk of all outcomes in individuals with PRISm and airflow limitation, irrespective of the metabolic syndrome (Fig. 2 (right part) and Supplementary Figures S11–S15). While the risk of ischemic heart disease/heart failure was slightly higher for those with PRISm compared to those with airflow limitation, the opposite was observed for respiratory disease. For all outcomes, the increased risk was driven by low lung function but also by the metabolic syndrome, and the highest risk was generally observed in individuals with both severe lung function impairment and the metabolic syndrome.

No multiplicative interactions between the metabolic syndrome, FEV_1_ Z-score, and outcome in individuals with PRISm or airflow limitation were found, suggesting that the association between lung function and outcome was similar in individuals with and without the metabolic syndrome. The additive model, however, indicated that FEV_1_ was a somewhat stronger predictor of outcome in those with the metabolic syndrome compared to those without the metabolic syndrome (Supplementary Figure S16).

Lung function (FEV_1_ Z-score) mediated similar proportions of the effects of the metabolic syndrome, HS-CRP, and fibrinogen on outcome and was especially high in individuals with airflow limitation (Supplementary Figure S17).

## Discussion

In the present study with more than 100,000 individuals from the Danish general population, we found that the metabolic syndrome conferred increased risk of cardiopulmonary morbidity and mortality at all levels of lung function impairment.

Mechanistically, the most likely explanation for why the metabolic syndrome confer increased risk of ischemic heart disease/heart failure comes from the various elements defining the metabolic syndrome, including elevated triglyceride- and cholesterol-rich remnant lipoproteins, a causal risk factor for atherosclerotic cardiovascular disease.^27^ For respiratory disease, it may be less straightforward; however, the metabolic syndrome is highly associated with low-grade inflammation and obesity, both important drivers of pulmonary disease.^12^^,^^15^^,^^28^^,^^29^ Finally, as the most common causes of death include ischemic heart disease/heart failure and respiratory disease, the increased all-cause mortality in those with versus without the metabolic syndrome reflects the increased risk of these two major diseases.

Airflow limitation and PRISm are physiologically defined based on spirometric findings. Both conditions are heterogeneous as they can be caused by different aetiologies and result from different pathogenic mechanisms involving the airways, lung parenchyma, chest wall, pleurae, and extrathoracic conditions, including abdominal obesity.^15^ In addition, in both airflow limitation and PRISm, multimorbidity is common.^30^^,^^31^ As reported previously,^32^, ^33^, ^34^ the present study underlines that individuals with the metabolic syndrome and poor lung function constitute a high-risk clinical group that needs special attention. In our analyses, we did not include the individual components of the metabolic syndrome but handled the syndrome as a composite measure. Yet, the single components of the metabolic syndrome, including dyslipidaemia, hyperglycaemia, hypertension, and obesity, are important to address individually if we wish to improve the prognosis of individuals with the metabolic syndrome and lung function impairment. A healthy lifestyle, including smoking avoidance, healthy nutrition, and sufficient physical activity, is important for both the control of cardiovascular risk factors and the preservation of lung function. Implementation of evidence-based treatments for hypertension and hypercholesterolaemia is also important. However, studies focusing on the management of patients with chronic lung diseases like COPD have shown a relatively low implementation of beta-blockers and statins, which leaves much room for improvement.^35^^,^^36^

Systemic inflammation has been linked with the aetiology of the metabolic syndrome, but the metabolic syndrome can also lead to increased inflammation and thereby create a vicious cycle that feeds upon itself.^29^ Previous studies of patients with COPD have identified persistent systemic inflammation as a marker of poor prognosis, including mortality.^12^^,^^37^ Systemic inflammation caused by smoking and/or inhalation of general pollutants has also been suggested to play a role in PRISm.^13^ Our results confirm a higher prevalence of individuals with elevated CRP and fibrinogen among those with airflow limitation and PRISm, particularly in the subgroups with the metabolic syndrome (Table 1). We also observed a strong positive association between systemic inflammation and both morbidity and mortality in individuals with PRISm and airflow limitation, irrespective of the metabolic syndrome and smoking status.

High body weight can affect respiration through a higher elastic loading of the muscles during inspiration, requiring greater effort to breathe.^29^ Deposits of abdominal and thoracic fat will mechanically have a negative effect on the movements of the diaphragm and chest wall, while fat in other areas of the body like the hips and thighs will not.^29^ Thus, abdominal obesity is likely to be the most important mechanism linking the metabolic syndrome and PRISm, as it has a greater impact on FVC than on FEV_1_, resulting in a restrictive pattern, which is characteristic of PRISm.^11^^,^^15^ Obesity in PRISm is an important condition to target in order to reduce both the increased cardiovascular risk and improve lung function.^11^^,^^15^ New therapies, including GLP-1 receptor analogues either alone or in combination with other gut hormones, have been shown to lead to significant weight loss associated with lung function improvement.^38^^,^^39^ High BMI has also been causally associated with increased severe exacerbations and pneumonias in individuals with COPD in the present cohort.^28^ Yet, the prognostic role of obesity in COPD may be more complex than in PRISm. Although severe obesity is an established risk factor for pulmonary complications,^40^ some studies suggest that individuals with COPD and increased BMI have a better prognosis than individuals with COPD and normal-range BMI, the so-called “obesity paradox”.^29^ In fact, the analysis of the association of the metabolic syndrome with mortality in COPD patients from the UK primary care showed that, whereas the presence of hypertension and type 2 diabetes mellitus increased mortality, obesity was associated with reduced mortality.^34^ Nevertheless, new observations of the effect of GLP-1 agonists in individuals with both type-2 diabetes and COPD suggest beneficial effects of weight loss, including a lower risk of severe exacerbations.^41^

The strengths of our study are the large and well-characterised general population sample, access to the all-covering Danish registries regarding morbidity and mortality without losses to follow-up, and the long follow-up. A potential limitation is that individuals were assigned into clinical groups of lung function impairment based on pre-bronchodilator measurements, which may limit our external generalizability of those with airflow limitation towards COPD, but this limitation is less important for those with PRISm. In general, a reversible form of airflow limitation could suggest the presence of asthma, but some patients with COPD also display a degree of reversibility in lung function.^31^ Spirometry was done at one point in time, which could lead to misclassification as subjects could move from one lung function phenotype to another during follow-up.^11^^,^^42^ PRISm, airflow limitation, and the metabolic syndrome share many of the same risk factors, and this could pose a problem for the assumption of no unmeasured exposure-mediator confounding in the mediation analyses. Waist circumference is a key variable in the definition of the metabolic syndrome, depending on sex and ethnicity. However, no gold standard exists, and different thresholds to define abdominal obesity have been proposed. The recommended thresholds for Europeans are either ≥94 cm for men and ≥80 cm for women, or ≥102 cm for men and ≥88 cm for women,^22^ and we chose the latter based on the distribution of waist circumference in our sample. Some individuals with the metabolic syndrome may have been misclassified as not having the syndrome because we did not have information about drugs used for elevated triglycerides and reduced HDL cholesterol, such as fibrates, nicotinic acid, and high-dose ω-3 fatty acids.^22^ These medications are rarely used in Denmark, and the misclassification will most likely bias associations towards the null. Although our study sample is not representative of the whole Danish population as it comprises a suburban population around the capital with a slightly higher social status than the country average, our findings of the importance of the metabolic syndrome for the prognosis of airflow limitation and PRISm are likely to be generalizable to the rest of Denmark and to other high-income countries.

In conclusion, in the general population of a high-income country, the presence of the metabolic syndrome was associated with increased cardiopulmonary morbidity and mortality in individuals with PRISm and airflow limitation across all levels of lung function impairment. The metabolic syndrome explained 13% of the influence of lung function impairment phenotype (PRISm or airflow limitation) on ischemic heart disease/heart failure and 27% on all-cause mortality.

## Contributors

J.L.M. and P.L. take full responsibility for the integrity of the data and the formal analysis. Conceptualisation, study design, and acquisition of data: J.L.M. and P.L. Analysis and interpretation of data: J.L.M., T.S.I., Y.Ç., H.K., P.S.B., J.V., B.G.N., and P.L. Writing–original draft: J.L.M. and P.L. Writing–review & editing: J.L.M., T.S.I., Y.Ç., H.K., P.S.B., J.V., B.G.N., and P.L. Statistical analysis and visualisation: J.L.M. Obtained funding: J.L.M., B.G.N., and P.L. Supervision: B.G.N. and P.L. All authors approved the final version submitted to the journal.

## Data sharing statement

Individual participant data used in this article will not be shared.

## Declaration of interests

J.L.M. received an unrestricted grant from The Danish Lung Foundation in relation to the present work. T.S.I. received an advisory board fee from AstraZeneca. Y.Ç. received speaking fees from Sanofi Genzyme, AstraZeneca, GlaxoSmithKline, and Boehringer Ingelheim. H.K. reports funding from The Swedish Science Council, The Swedish Heart and Lung Foundation, The Swedish Asthma and Allergy Foundation, The Tampere Tuberculosis Foundation, The Swedish ALF-funding, and The Finnish Anti-Tuberculosis Association Foundation outside the submitted work; being a committee member of the research council of the Swedish Heart and Lung Foundation; consulting fees from GlaxoSmithKline, AstraZeneca, MSD, Novartis, Orion Pharma, and Sanofi Genzyme; and speaking fees from AstraZeneca, Boehringer Ingelheim, GlaxoSmithKline, and Orion Pharma. P.S.B. received lecture fees from AstraZeneca and Boehringer Ingelheim and advisory board fees from GlaxoSmithKline and AstraZeneca. J.V. received consulting fees from ALK, AstraZeneca, Boehringer Ingelheim, GlaxoSmithKline, and Teva, as well as speaking fees from AstraZeneca, Boehringer Ingelheim, Chiesi, and GlaxoSmithKline and advisory board fees from AstraZeneca. P.L. received an unrestricted grant in relation to the present work from Boehringer Ingelheim; advisory board fees from AstraZeneca, GlaxoSmithKline, Boehringer Ingelheim, and Sanofi; and speaking fees from AstraZeneca, Boehringer Ingelheim, and GlaxoSmithKline. B.G.N. declares no potential conflicts of interest.
