# Supplementary material for: Impact of the metabolic syndrome on cardiopulmonary morbidity and mortality in individuals with lung function impairment: a prospective cohort study of the Danish general population
Source: Lancet Reg Health Eur. 2023 Nov 6;35:100759. doi: 10.1016/j.lanepe.2023.100759 (PMC10652137; doi:10.1016/j.lanepe.2023.100759)
Supplement: Supplementary data and Tables S1 and S2 and Figures S1–S17 [file mmc1.docx]

**Supplementary data**

**Impact of the metabolic syndrome on cardiopulmonary morbidity and mortality in individuals with lung function impairment: a prospective cohort study of the Danish general population**

Jacob Louis Marott, Truls Sylvan Ingebrigtsen, Yunus Çolak, Hannu Kankaanranta, Per Sigvald Bakke, Jørgen Vestbo, Børge Grønne Nordestgaard, Peter Lange

**Supplementary Table 1.** Baseline characteristics in PRISm according to lung function impairment stages and presence of the metabolic syndrome. **Page 3+4**

**Supplementary Table 2.** Baseline characteristics in airflow limitation according to lung function impairment stages and presence of the metabolic syndrome. **Page 5+6**

**Supplementary Figure 1.** Definition of the lung function phenotypes and the severity staging of lung function impairment. **Page 7**

**Supplementary Figure 2.** Joint distribution of the metabolic syndrome, smoking status, and presence of asthma in normal lung function, PRISm, and airflow limitation. **Page 8**

**Supplementary Figure 3.** Risk of ischemic heart disease or heart failure morbidity in normal lung function, PRISm, and airflow limitation with and without presence of the metabolic syndrome. **Page 9**

**Supplementary Figure 4.** Risk of respiratory disease morbidity in normal lung function, PRISm, and airflow limitation with and without presence of the metabolic syndrome. **Page 10**

**Supplementary Figure 5.** Risk of all-cause mortality in normal lung function, PRISm, and airflow limitation with and without presence of the metabolic syndrome. **Page 11**

**Supplementary Figure 6.** Risk of cardiac mortality in normal lung function, PRISm, and airflow limitation with and without presence of the metabolic syndrome. **Page 12**

**Supplementary Figure 7.** Risk of respiratory disease mortality in normal lung function, PRISm, and airflow limitation with and without presence of the metabolic syndrome. **Page 13**

**Supplementary Figure 8.** Multiplicative and additive moderation analysis in all individuals. **Page 14**

**Supplementary Figure 9.** Mediation analysis for HS-CRP. **Page 15**

**Supplementary Figure 10.** Mediation analysis for fibrinogen. **Page 16**

**Supplementary Figure 11.** Cumulative incidence of ischemic heart disease or heart failure morbidity. **Page 17**

**Supplementary Figure 12.** Cumulative incidence of respiratory disease morbidity. **Page 18**

**Supplementary Figure 13.** Cumulative incidence of all-cause mortality. **Page 19**

**Supplementary Figure 14.** Cumulative incidence of cardiac mortality. **Page 20**

**Supplementary Figure 15.** Cumulative incidence of respiratory disease mortality. **Page 21**

**Supplementary Figure 16.** Multiplicative and additive moderation analysis in PRISm and airflow limitation. **Page 22**

**Supplementary Figure 17.** Mediation analysis for lung function. **Page 23**

| **Supplementary Table 1.** Baseline characteristics according to the metabolic syndrome and severity stages of lung function impairment in PRISm in 6,126 individuals from the Copenhagen General Population Study. | | | | | | |
| --- | --- | --- | --- | --- | --- | --- |
|  | **PRISm** (n=6,126) | | | | | |
|  | **Mild**  **Without MetS**  (n=3,100) | **Mild**  **With MetS**  (n=1,919) | **Moderate**  **Without MetS**  (n=525) | **Moderate**  **With MetS**  (n=384) | **Severe**  **Without MetS**  (n=101) | **Severe**  **With MetS**  (n=97) |
| *General characteristics* |  |  |  |  |  |  |
| Male sex – no. (%) | 1,188 (38) | 1,004 (52) | 188 (36) | 178 (46) | 31 (31) | 52 (54) |
| Age – years | 57±12 | 60±11 | 58±12 | 59±11 | 58±11 | 59±10 |
| FEV_1_ |  |  |  |  |  |  |
| Mean – L | 2·3±0·6 | 2·3±0·6 | 1·9±0·5 | 2·0±0·6 | 1·5±0·5 | 1·6±0·6 |
| Percent of predicted value | 73±6 | 72±5 | 62±7 | 62±6 | 49±10 | 49±9 |
| Z-score | -2·0±0·2 | -2·0±0·2 | -2·8±0·2 | -2·8±0·2 | -3·8±0·5 | -3·7±0·5 |
| FVC |  |  |  |  |  |  |
| Mean – L | 3·0±0·8 | 3·0±0·8 | 2·5±0·7 | 2·6±0·7 | 1·9±0·6 | 2·1±0·7 |
| Percent of predicted value | 76±7 | 74±7 | 65±8 | 64±8 | 50±12 | 51±10 |
| Z-score | -1·7±0·4 | -1·8±0·4 | -2·6±0·4 | -2·6±0·4 | -3·6±0·7 | -3·6±0·6 |
| FEV_1_/FVC | 0·76±0·06 | 0·77±0·06 | 0·76±0·06 | 0·76±0·06 | 0·78±0·08 | 0·77±0·07 |
| *Symptoms* |  |  |  |  |  |  |
| Dyspnoea (mMRC ≥2) – no. (%) | 409 (13) | 523 (27) | 133 (26) | 127 (33) | 31 (31) | 49 (51) |
| Chronic mucus hypersecretion – no. (%) | 383 (12) | 312 (16) | 92 (18) | 75 (20) | 22 (22) | 28 (29) |
| Frequent exacerbations – no. (%) | 111 (4) | 81 (4) | 28 (5) | 29 (8) | 11 (11) | 12 (13) |
| Exposure to dust/fumes – no. (%) | 361 (12) | 392 (21) | 81 (15) | 83 (22) | 13 (13) | 22 (23) |
| Wheezing – no. (%) | 852 (28) | 772 (41) | 187 (36) | 171 (45) | 40 (40) | 50 (54) |
| *Lifestyle factors* |  |  |  |  |  |  |
| Body mass index |  |  |  |  |  |  |
| Mean – kg/m^2^ | 26±4 | 31±5 | 26±5 | 31±6 | 25±5 | 32±7 |
| ≥25 – no. (%) | 1,586 (51) | 1,785 (93) | 279 (53) | 340 (89) | 49 (49) | 85 (88) |
| ≥30 – no. (%) | 412 (13) | 1,020 (53) | 94 (18) | 206 (54) | 13 (13) | 54 (56) |
| Waist circumference – cm | 89±13 | 106±13 | 91±14 | 107±13 | 89±14 | 110±16 |
| Smoking history |  |  |  |  |  |  |
| Never-smoker – no. (%) | 982 (33) | 522 (28) | 158 (31) | 76 (21) | 38 (39) | 22 (24) |
| Former smoker – no. (%) | 1,140 (38) | 743 (40) | 187 (37) | 143 (40) | 22 (22) | 38 (42) |
| Current smoker – no. (%) | 855 (29) | 590 (32) | 164 (32) | 143 (40) | 38 (39) | 31 (34) |
| Consumption in pack-years, median [IQR] | 23 [10—36] | 30 [15—46] | 26 [12—39] | 31 [16—44] | 26 [17—37] | 32 [18—48] |
| Alcohol intake – no. (%) |  |  |  |  |  |  |
| Never | 261 (9) | 229 (13) | 57 (12) | 52 (15) | 12 (13) | 15 (19) |
| Moderate | 2,046 (71) | 1,165 (66) | 332 (69) | 227 (64) | 67 (73) | 46 (58) |
| High | 570 (20) | 363 (21) | 93 (19) | 76 (21) | 13 (14) | 18 (23) |
| Physical activity – no. (%) |  |  |  |  |  |  |
| Low | 274 (9) | 304 (16) | 58 (11) | 66 (18) | 16 (16) | 21 (22) |
| Moderate | 1,496 (49) | 1,010 (53) | 278 (54) | 206 (55) | 58 (59) | 49 (52) |
| High | 1,294 (42) | 582 (31) | 176 (34) | 105 (28) | 24 (24) | 24 (26) |
| Education – no. (%) |  |  |  |  |  |  |
| <Middle school | 341 (11) | 326 (17) | 73 (14) | 75 (20) | 16 (16) | 20 (21) |
| Middle school | 1,384 (45) | 965 (51) | 260 (50) | 213 (56) | 47 (47) | 54 (56) |
| High school | 971 (31) | 479 (25) | 152 (29) | 71 (19) | 28 (28) | 17 (18) |
| University | 390 (13) | 140 (7) | 39 (7) | 23 (6) | 9 (9) | 5 (5) |
| *Comorbidities and clinical measurements* |  |  |  |  |  |  |
| Current asthma – no. (%) | 270 (9) | 183 (10) | 51 (10) | 52 (14) | 12 (12) | 12 (13) |
| Asthma as child – no. (%) | 438 (14) | 259 (14) | 80 (15) | 39 (10) | 11 (11) | 10 (11) |
| Diabetes – no. (%) | 78 (3) | 307 (16) | 14 (3) | 76 (20) | 3 (3) | 21 (22) |
| Blood pressure – mmHg |  |  |  |  |  |  |
| Systolic | 140±22 | 150±21 | 139±22 | 147±20 | 138±25 | 147±22 |
| Diastolic | 83±12 | 88±12 | 83±12 | 87±13 | 82±14 | 87±12 |
| Blood pressure medication – no. (%) | 551 (19) | 668 (37) | 108 (22) | 132 (36) | 18 (19) | 39 (40) |
| *Blood samples* |  |  |  |  |  |  |
| Glucose >7·8 mmol/L – no. (%) | 43 (1) | 226 (12) | 2 (0) | 51 (13) | 4 (4) | 11 (11) |
| HDL cholesterol <1·3 (men) / 1·03 (women) mmol/L – no. (%) | 223 (7) | 1,043 (54) | 46 (9) | 223 (58) | 9 (9) | 53 (55) |
| Triglycerides ≥1·7 mmol/L – no. (%) | 680 (22) | 1,709 (89) | 111 (21) | 340 (89) | 30 (30) | 83 (86) |
| Fibrinogen ≥14 µmol/L – no. (%) | 584 (19) | 508 (27) | 145 (28) | 120 (31) | 28 (28) | 47 (48) |
| HS-CRP ≥3 mg/L – no. (%) | 744 (24) | 873 (46) | 174 (33) | 203 (53) | 39 (39) | 61 (63) |
| IgE ≥150 IU/mL – no. (%) | 117 (11) | 100 (14) | 21 (13) | 20 (14) | 3 (7) | 6 (19) |
| Eosinophils ≥500 cells/µL – no. (%) | 101 (3) | 81 (4) | 16 (3) | 24 (6) | 4 (4) | 2 (2) |
| Data are n (%), mean±SD, or median [IQR]. Abbreviations: PRISm, preserved ratio impaired spirometry; MetS, metabolic syndrome; FEV_1_, forced expiratory volume in 1 second; FVC, forced vital capacity; mMRC, modified Medical Research Council dyspnoea scale; IQR, interquartile range; HDL, high-density lipoprotein; HS-CRP, high-sensitivity C-reactive protein; IgE, immunoglobulin E. | | | | | | |

| **Supplementary Table 2.** Baseline characteristics according to the metabolic syndrome and severity stages of lung function impairment in airflow limitation in 14,560 individuals from the Copenhagen General Population Study. | | | | | | | | |
| --- | --- | --- | --- | --- | --- | --- | --- | --- |
|  | **Airflow limitation** (n=14,560) | | | | | | | |
|  | **Very mild**  **Without MetS**  (n=7,112) | **Very mild**  **With MetS**  (n=1,582) | **Mild**  **Without MetS**  (n=2,295) | **Mild**  **With MetS**  (n=846) | **Moderate**  **Without MetS**  (n=1,115) | **Moderate**  **With MetS**  (n=513) | **Severe**  **Without MetS**  (n=741) | **Severe**  **With MetS**  (n=356) |
| *General characteristics* |  |  |  |  |  |  |  |  |
| Male sex – no. (%) | 3,272 (46) | 880 (56) | 1,057 (46) | 494 (58) | 548 (49) | 309 (60) | 331 (45) | 198 (56) |
| Age – years | 60±13 | 62±13 | 62±13 | 64±11 | 63±12 | 64±10 | 63±10 | 63±10 |
| FEV_1_ |  |  |  |  |  |  |  |  |
| Mean – L | 2.8±0.8 | 2.7±0.8 | 2.1±0.6 | 2.1±0.6 | 1.7±0.6 | 1.8±0.5 | 1.3±0.5 | 1.3±0.5 |
| Percent of predicted value | 92±11 | 89±11 | 70±7 | 69±7 | 58±8 | 58±7 | 43±10 | 42±10 |
| Z-score | -0.6±0.7 | -0.7±0.7 | -2.0±0.2 | -2.1±0.2 | -2.9±0.2 | -2.9±0.2 | -4.0±0.6 | -4.0±0.7 |
| FVC |  |  |  |  |  |  |  |  |
| Mean – L | 4.3±1.1 | 4.2±1.1 | 3.4±0.9 | 3.4±0.9 | 3.0±0.9 | 3.0±0.8 | 2.5±0.8 | 2.5±0.8 |
| Percent of predicted value | 111±14 | 109±14 | 89±11 | 87±9 | 79±12 | 76±11 | 66±14 | 63±14 |
| Z-score | 0.8±1.0 | 0.6±0.9 | -0.7±0.7 | -0.9±0.6 | -1.4±0.8 | -1.6±0.6 | -2.3±0.9 | -2.6±0.9 |
| FEV_1_/FVC | 0·65±0·05 | 0·65±0·05 | 0·62±0·07 | 0·63±0·06 | 0·58±0·08 | 0·60±0·07 | 0·51±0·10 | 0·54±0·10 |
| *Symptoms* |  |  |  |  |  |  |  |  |
| Dyspnoea (mMRC ≥2) – no. (%) | 424 (6) | 221 (14) | 391 (17) | 205 (24) | 293 (26) | 188 (37) | 345 (47) | 193 (55) |
| Chronic mucus hypersecretion – no. (%) | 779 (11) | 220 (14) | 463 (20) | 187 (22) | 297 (27) | 147 (29) | 301 (41) | 149 (42) |
| Frequent exacerbations – no. (%) | 149 (2) | 39 (2) | 113 (5) | 63 (8) | 104 (9) | 47 (9) | 124 (17) | 66 (19) |
| Exposure to dust/fumes – no. (%) | 654 (9) | 240 (15) | 328 (14) | 153 (18) | 225 (20) | 135 (26) | 200 (27) | 103 (29) |
| Wheezing – no. (%) | 1,376 (20) | 450 (29) | 829 (37) | 396 (47) | 545 (50) | 281 (56) | 503 (69) | 246 (71) |
| *Lifestyle factors* |  |  |  |  |  |  |  |  |
| Body mass index |  |  |  |  |  |  |  |  |
| Mean – kg/m^2^ | 25±3 | 29±4 | 25±4 | 29±4 | 25±4 | 30±5 | 24±4 | 30±5 |
| ≥25 – no. (%) | 2,814 (40) | 1,339 (85) | 977 (43) | 725 (86) | 469 (42) | 447 (87) | 288 (39) | 303 (85) |
| ≥30 – no. (%) | 374 (5) | 519 (33) | 186 (8) | 333 (39) | 91 (8) | 216 (42) | 70 (9) | 141 (40) |
| Waist circumference – cm | 86±10 | 100±10 | 88±12 | 103±12 | 89±12 | 105±12 | 89±13 | 106±13 |
| Smoking history |  |  |  |  |  |  |  |  |
| Never-smoker – no. (%) | 2,169 (32) | 373 (24) | 456 (21) | 123 (15) | 160 (15) | 52 (10) | 66 (9) | 30 (8) |
| Former smoker – no. (%) | 2,997 (44) | 761 (50) | 912 (41) | 400 (49) | 485 (45) | 246 (49) | 306 (43) | 160 (45) |
| Current smoker – no. (%) | 1,713 (25) | 391 (26) | 853 (38) | 298 (36) | 437 (40) | 199 (40) | 339 (48) | 164 (46) |
| Consumption in pack-years, median [IQR] | 20 [8—34] | 25 [13—40] | 28 [15—42] | 32 [19—48] | 33 [20—47] | 38 [23—53] | 37 [23—50] | 40 [27—55] |
| Alcohol intake – no. (%) |  |  |  |  |  |  |  |  |
| Never | 514 (7) | 162 (11) | 207 (9) | 104 (13) | 120 (11) | 64 (13) | 75 (11) | 41 (13) |
| Moderate | 5,016 (73) | 1,063 (70) | 1,493 (68) | 506 (64) | 653 (63) | 291 (60) | 425 (63) | 195 (60) |
| High | 1,376 (20) | 296 (19) | 484 (22) | 185 (23) | 271 (26) | 131 (27) | 173 (26) | 87 (27) |
| Physical activity – no. (%) |  |  |  |  |  |  |  |  |
| Low | 367 (5) | 167 (11) | 180 (8) | 105 (13) | 116 (11) | 90 (18) | 98 (13) | 92 (27) |
| Moderate | 2,742 (39) | 741 (47) | 1,051 (46) | 414 (49) | 533 (48) | 264 (52) | 406 (56) | 169 (49) |
| High | 3,947 (56) | 659 (42) | 1,040 (46) | 320 (38) | 451 (41) | 150 (30) | 227 (31) | 86 (25) |
| Education – no. (%) |  |  |  |  |  |  |  |  |
| <Middle school | 703 (10) | 215 (14) | 327 (14) | 167 (20) | 198 (18) | 114 (22) | 169 (23) | 94 (26) |
| Middle school | 2,696 (38) | 722 (46) | 1,020 (45) | 407 (48) | 526 (47) | 254 (50) | 352 (48) | 171 (48) |
| High school | 2,391 (34) | 437 (28) | 617 (27) | 185 (22) | 288 (26) | 93 (18) | 169 (23) | 71 (20) |
| University | 1,294 (18) | 200 (13) | 315 (14) | 82 (10) | 100 (9) | 49 (10) | 45 (6) | 19 (5) |
| *Comorbidities and clinical measurements* |  |  |  |  |  |  |  |  |
| Current asthma – no. (%) | 662 (9) | 151 (10) | 432 (19) | 122 (15) | 245 (22) | 117 (23) | 237 (33) | 119 (34) |
| Asthma as child – no. (%) | 1,097 (16) | 215 (14) | 402 (18) | 117 (14) | 218 (20) | 73 (14) | 124 (17) | 55 (15) |
| Diabetes – no. (%) | 116 (2) | 167 (11) | 55 (2) | 99 (12) | 38 (3) | 84 (17) | 16 (2) | 68 (19) |
| Blood pressure – mmHg |  |  |  |  |  |  |  |  |
| Systolic | 140 (21) | 149 (19) | 141 (22) | 149 (20) | 143 (22) | 150 (20) | 142 (22) | 149 (22) |
| Diastolic | 83 (11) | 87 (11) | 83 (12) | 87 (11) | 84 (12) | 86 (12) | 84 (12) | 86 (13) |
| Blood pressure medication – no. (%) | 1,121 (18) | 474 (35) | 460 (22) | 279 (36) | 238 (23) | 196 (41) | 154 (23) | 129 (39) |
| *Blood samples* |  |  |  |  |  |  |  |  |
| Glucose >7·8 mmol/L – no. (%) | 85 (1) | 130 (8) | 37 (2) | 74 (9) | 20 (2) | 60 (12) | 12 (2) | 47 (13) |
| HDL cholesterol <1·3 (men) / 1·03 (women) mmol/L – no. (%) | 503 (7) | 904 (57) | 152 (7) | 442 (52) | 67 (6) | 257 (50) | 41 (6) | 161 (45) |
| Triglycerides ≥1·7 mmol/L – no. (%) | 1,405 (20) | 1,395 (88) | 475 (21) | 756 (89) | 266 (24) | 446 (87) | 145 (20) | 313 (88) |
| Fibrinogen ≥14 µmol/L – no. (%) | 615 (9) | 237 (15) | 367 (16) | 166 (20) | 263 (24) | 128 (25) | 228 (31) | 127 (36) |
| HS-CRP ≥3 mg/L – no. (%) | 954 (14) | 397 (26) | 486 (21) | 292 (35) | 299 (27) | 225 (44) | 248 (34) | 186 (52) |
| IgE ≥150 IU/mL – no. (%) | 450 (12) | 120 (14) | 196 (18) | 64 (16) | 106 (22) | 47 (21) | 50 (17) | 31 (23) |
| Eosinophils ≥500 cells/µL – no. (%) | 264 (4) | 60 (4) | 106 (5) | 59 (7) | 46 (4) | 35 (7) | 51 (7) | 24 (7) |
| Data are n (%), mean±SD, or median [IQR]. Abbreviations: MetS, metabolic syndrome; FEV_1_, forced expiratory volume in 1 second; FVC, forced vital capacity; mMRC, modified Medical Research Council dyspnoea scale; IQR, interquartile range; HDL, high-density lipoprotein; HS-CRP, high-sensitivity C-reactive protein; IgE, immunoglobulin E. | | | | | | | | |

**Supplementary Figure 1.** Definition of the lung function phenotypes (normal lung function, PRISm and airflow limitation) and the severity staging of the lung function impairment (very mild (only airflow limitation), mild, moderate, and severe).

**
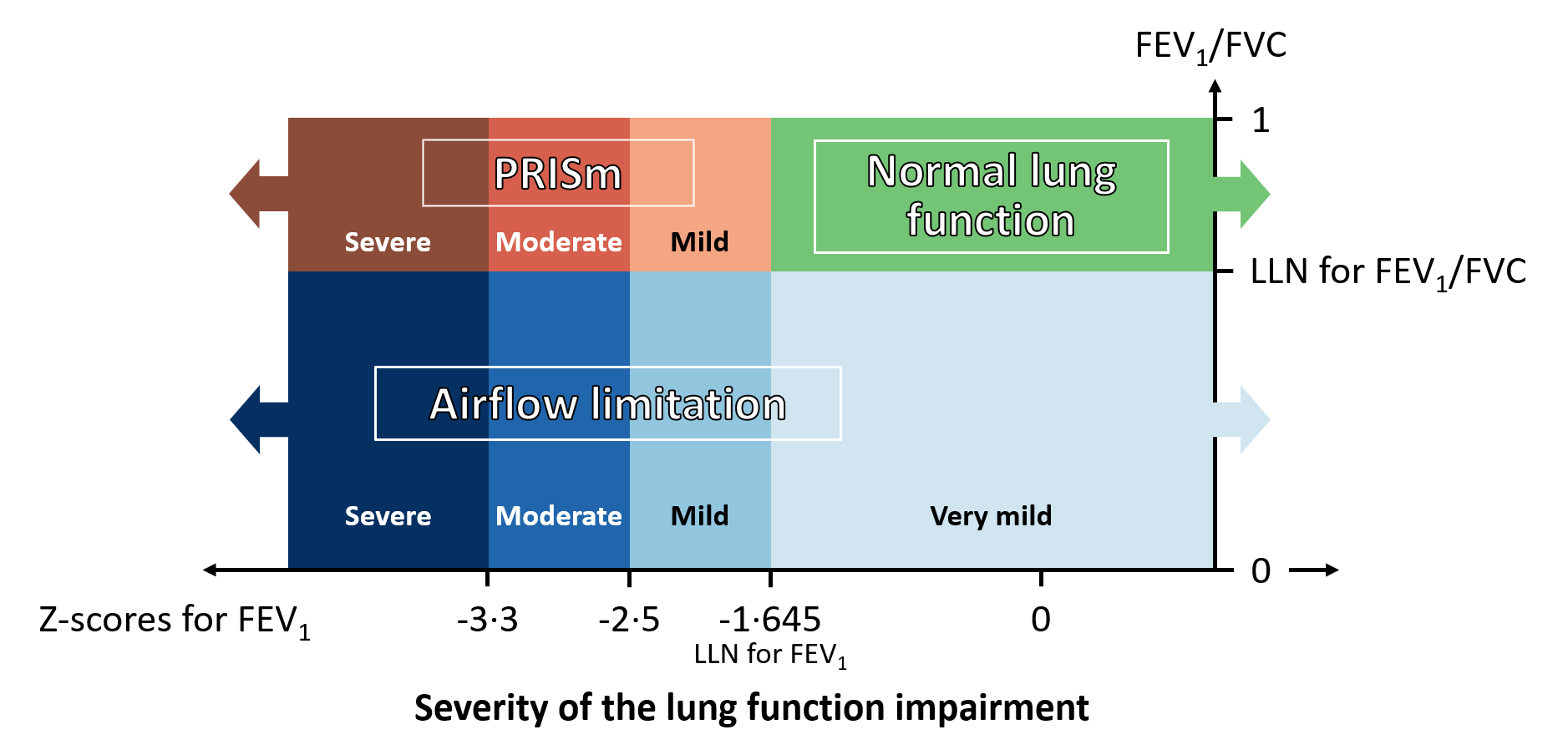
**

Abbreviations: PRISm, preserved ratio impaired spirometry; FEV_1_, forced expiratory volume in 1 second; FVC, forced vital capacity; LLN, lower limit of normal.

**Supplementary Figure 2.** Joint distribution of the metabolic syndrome, smoking status, and asthma in individuals with normal lung function (Panel A), PRISm (Panel B), and airflow limitation (Panel C). Asthma prevalence is depicted by triangles and black text.


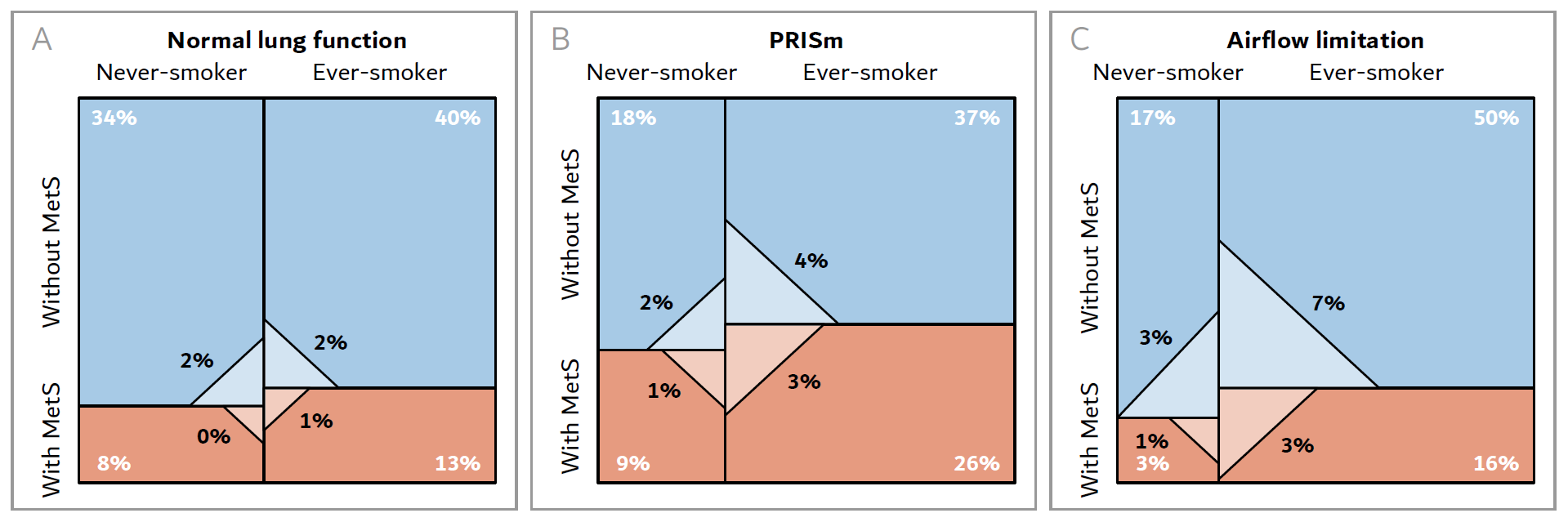


Abbreviations: PRISm, preserved ratio impaired spirometry; MetS, metabolic syndrome.

**Supplementary Figure 3.** Risk of ischemic heart disease or heart failure morbidity according to lung function phenotype and the metabolic syndrome with adjustment for age, sex, asthma, and smoking.


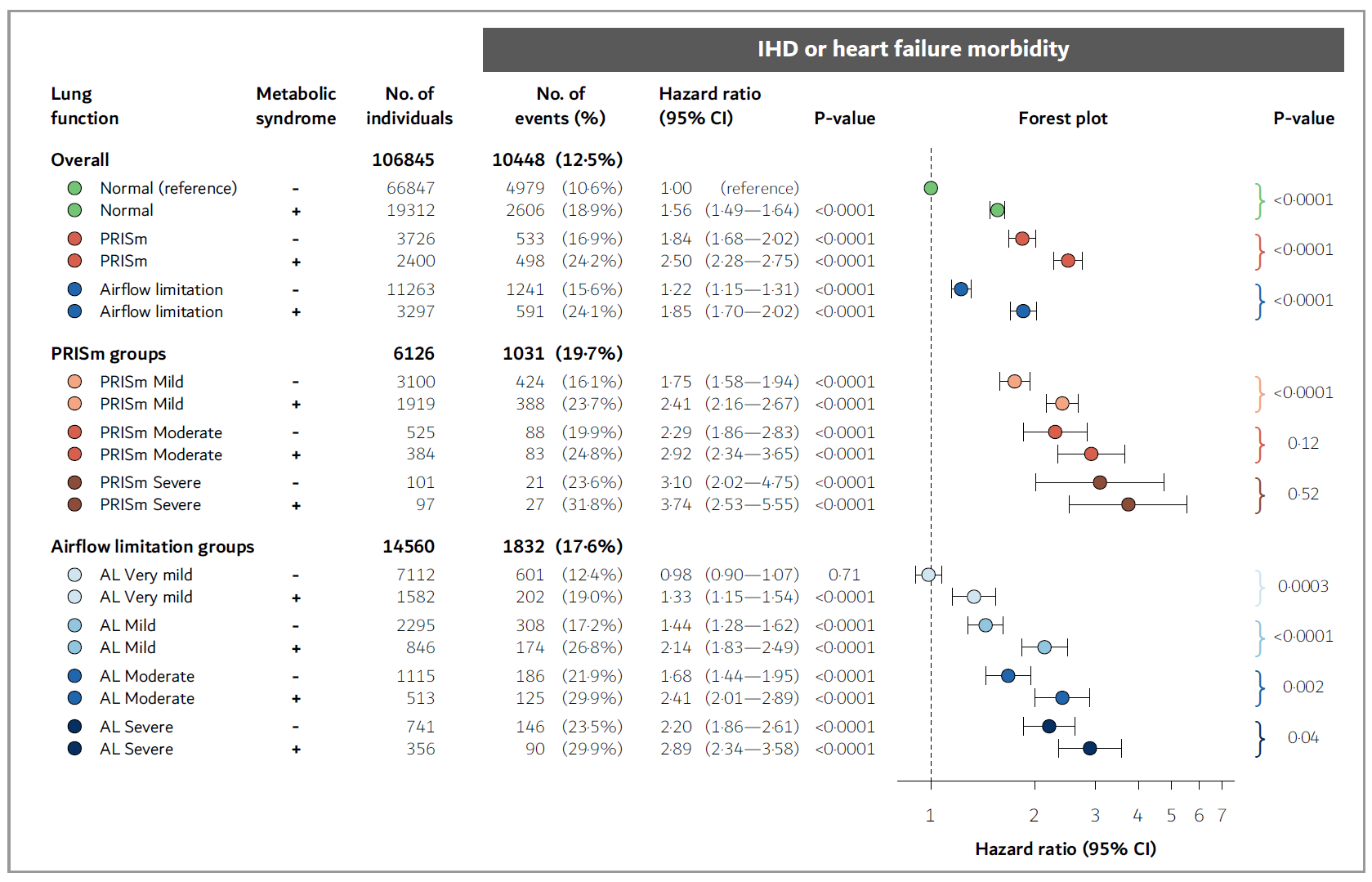


Abbreviations: PRISm, preserved ratio impaired spirometry; AL, airflow limitation; IHD, ischemic heart disease; CI, confidence interval; FEV_1_, forced expiratory volume in 1 second. The severity stages of the lung function impairment were defined according to FEV_1_ Z-scores.

**Supplementary Figure 4.** Risk of respiratory disease morbidity according to lung function phenotype and the metabolic syndrome with adjustment for age, sex, asthma, and smoking.


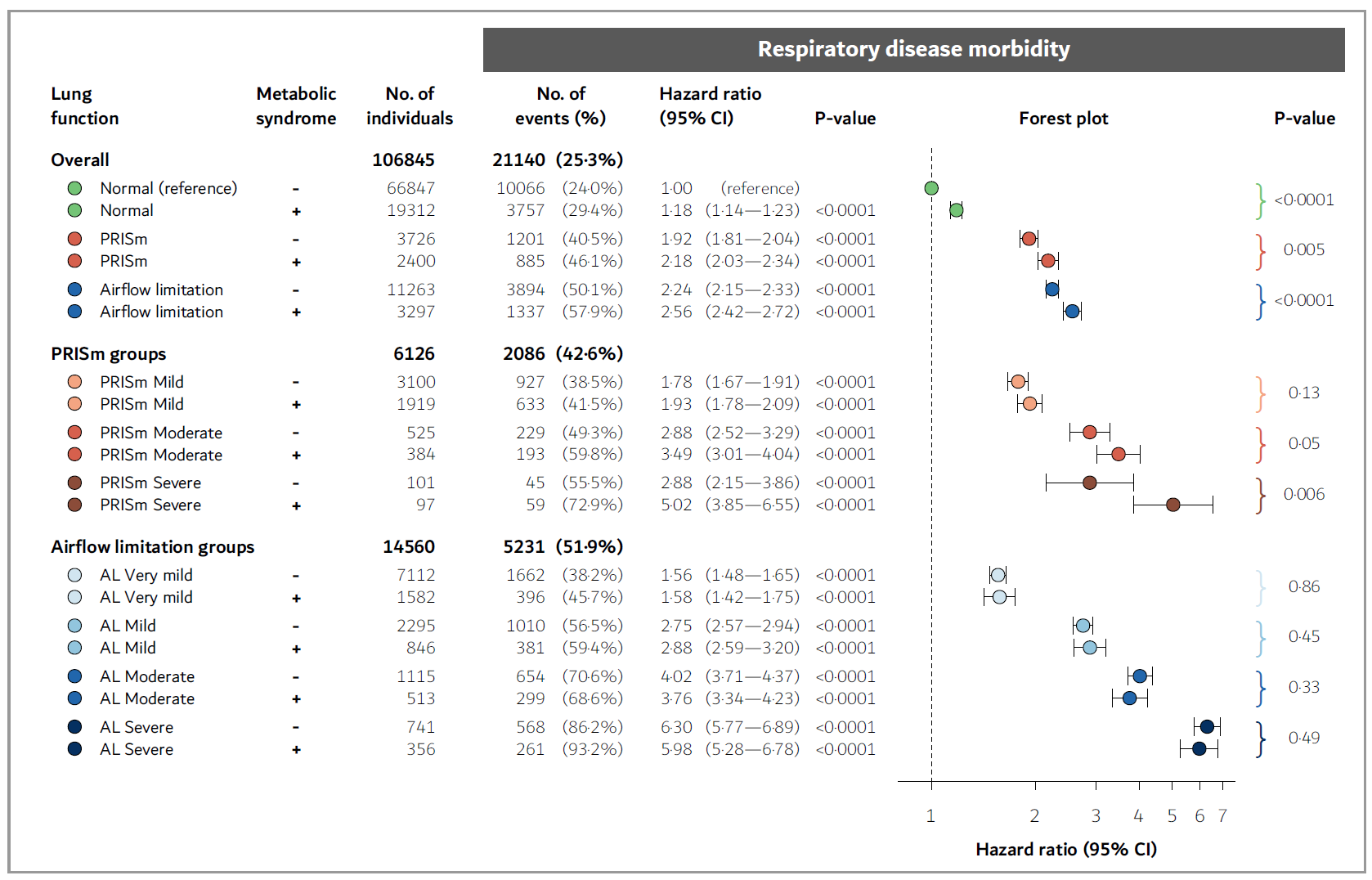


Abbreviations: PRISm, preserved ratio impaired spirometry; AL, airflow limitation; CI, confidence interval; FEV_1_, forced expiratory volume in 1 second. The severity stages of the lung function impairment were defined according to FEV_1_ Z-scores.

**Supplementary Figure 5.** Risk of all-cause mortality according to lung function phenotype and the metabolic syndrome with adjustment for age, sex, asthma, and smoking.


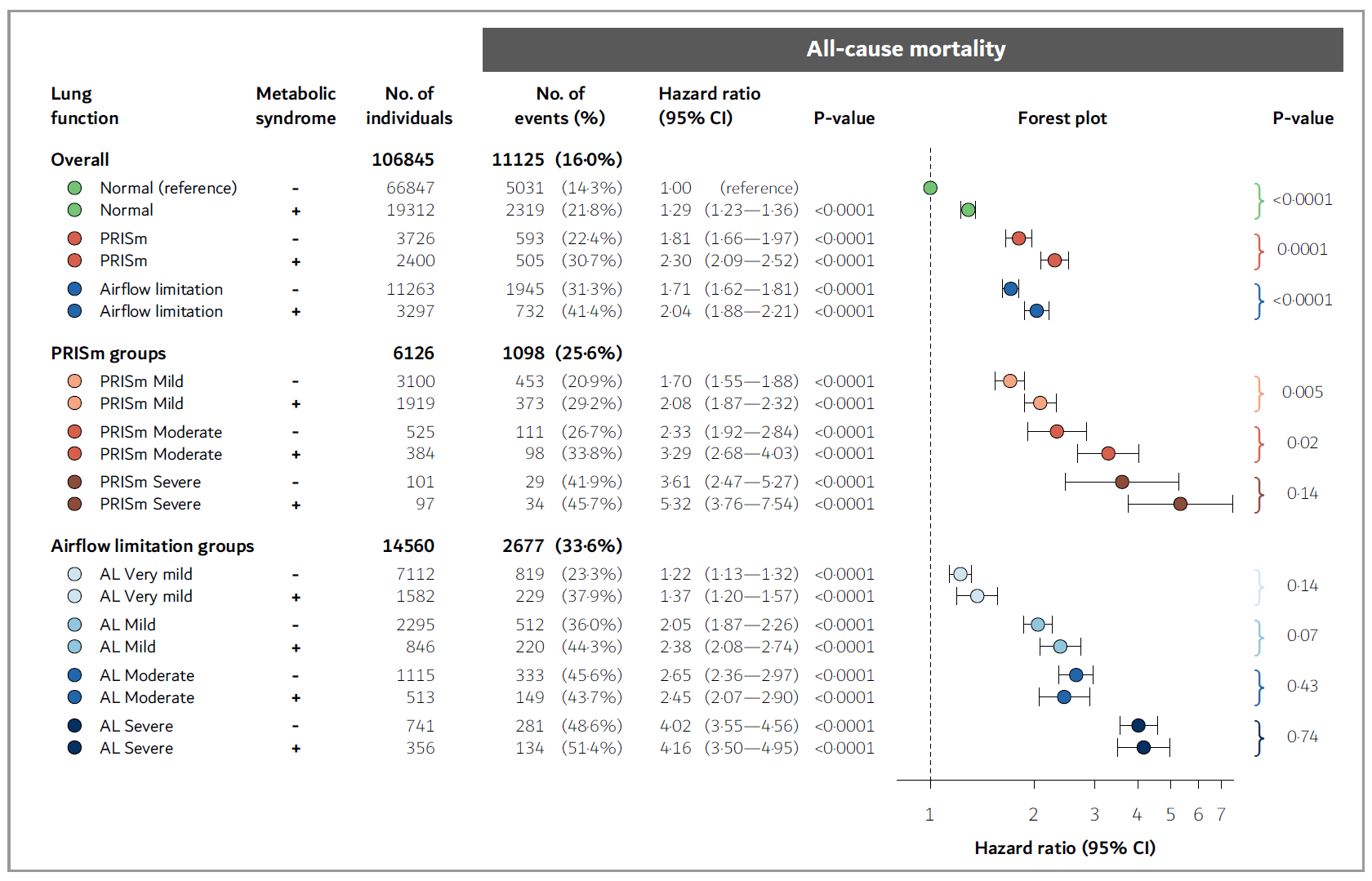


Abbreviations: PRISm, preserved ratio impaired spirometry; AL, airflow limitation; CI, confidence interval; FEV_1_, forced expiratory volume in 1 second. The severity stages of the lung function impairment were defined according to FEV_1_ Z-scores.

**Supplementary Figure 6.** Risk of cardiac mortality according to lung function phenotype and the metabolic syndrome with adjustment for age, sex, asthma, and smoking.


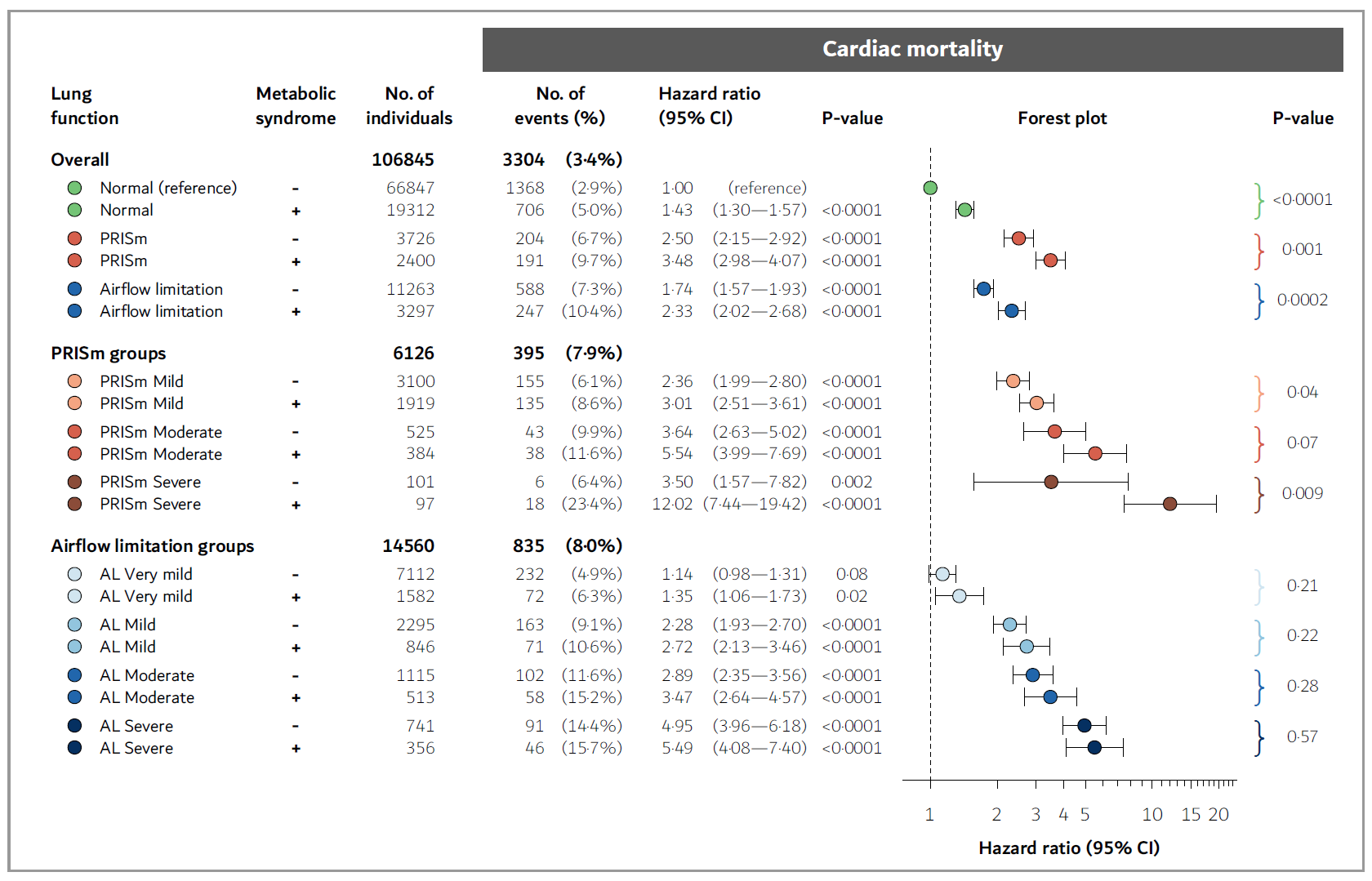


Abbreviations: PRISm, preserved ratio impaired spirometry; AL, airflow limitation; CI, confidence interval; FEV_1_, forced expiratory volume in 1 second. The severity stages of the lung function impairment were defined according to FEV_1_ Z-scores.

**Supplementary Figure 7.** Risk of respiratory disease mortality according to lung function phenotype and the metabolic syndrome with adjustment for age, sex, asthma, and smoking.


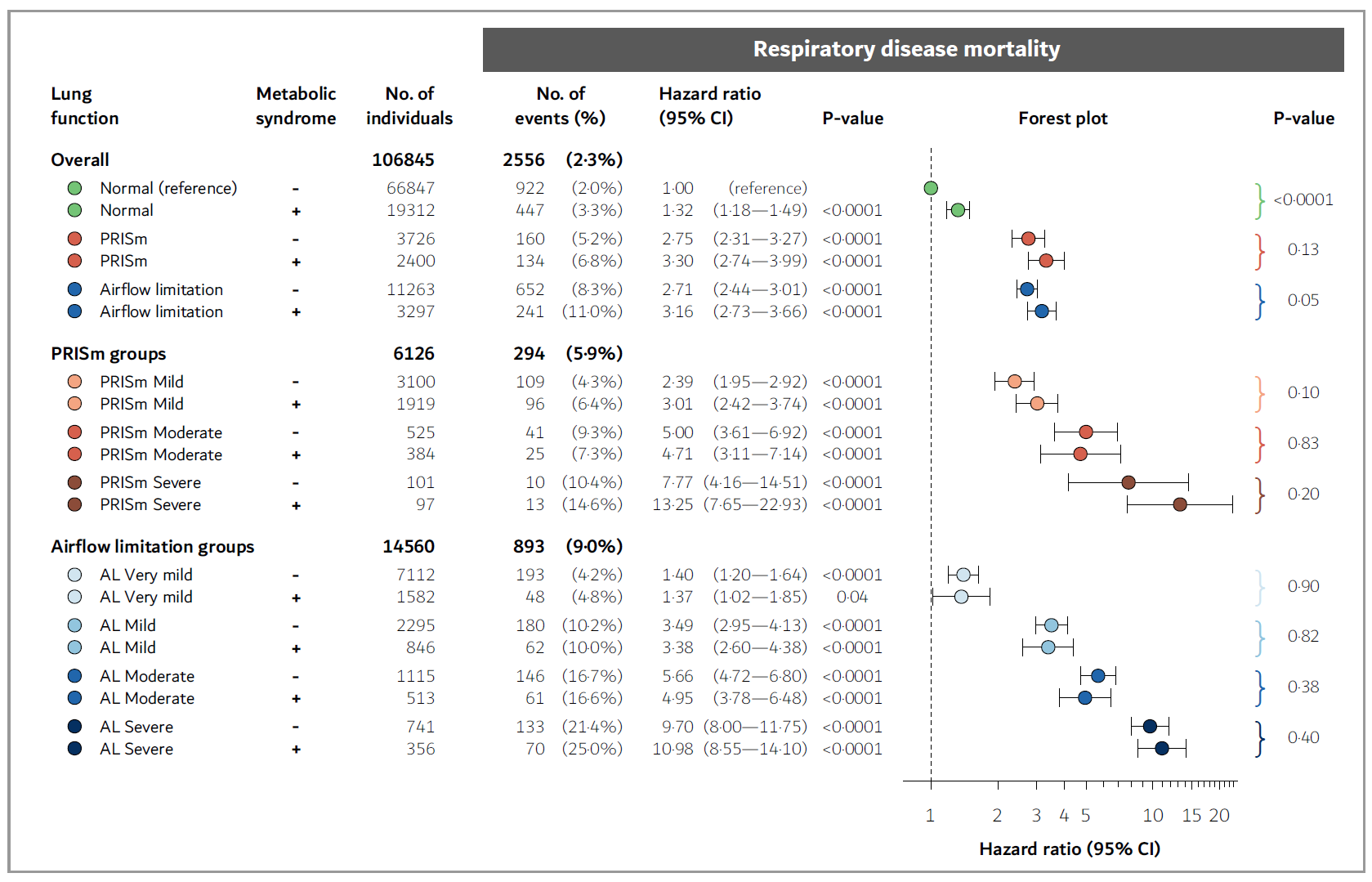


Abbreviations: PRISm, preserved ratio impaired spirometry; AL, airflow limitation; CI, confidence interval; FEV_1_, forced expiratory volume in 1 second. The severity stages of the lung function impairment were defined according to FEV_1_ Z-scores.

**Supplementary Figure 8.** Multiplicative and additive moderation analysis of lung function phenotype and the metabolic syndrome in ischemic heart disease or heart failure morbidity, respiratory disease morbidity, and all-cause mortality with adjustment for age, sex, asthma, and smoking. Normal lung function without the metabolic syndrome was chosen as reference group.


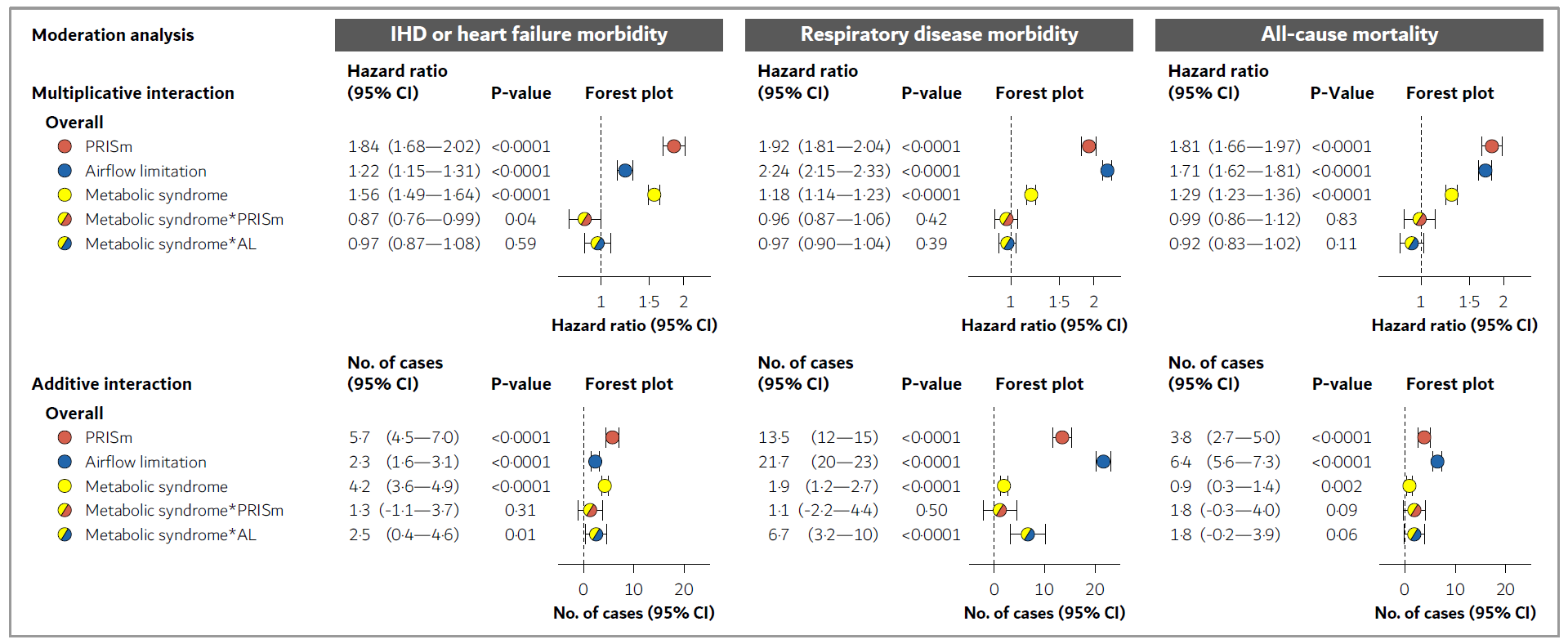


Abbreviations: PRISm, preserved ratio impaired spirometry; IHD, ischemic heart disease; CI, confidence interval. In the additive model, the estimated no. of additional cases is for each outcome presented per 1,000 person years.

**Supplementary Figure 9.** Mediation analysis between the exposure lung function (both lung function impairment phenotype and FEV_1_ Z-score), the mediator high-sensitivity C-reactive protein, and the outcomes ischemic heart disease or heart failure morbidity, respiratory disease morbidity, and all-cause mortality.


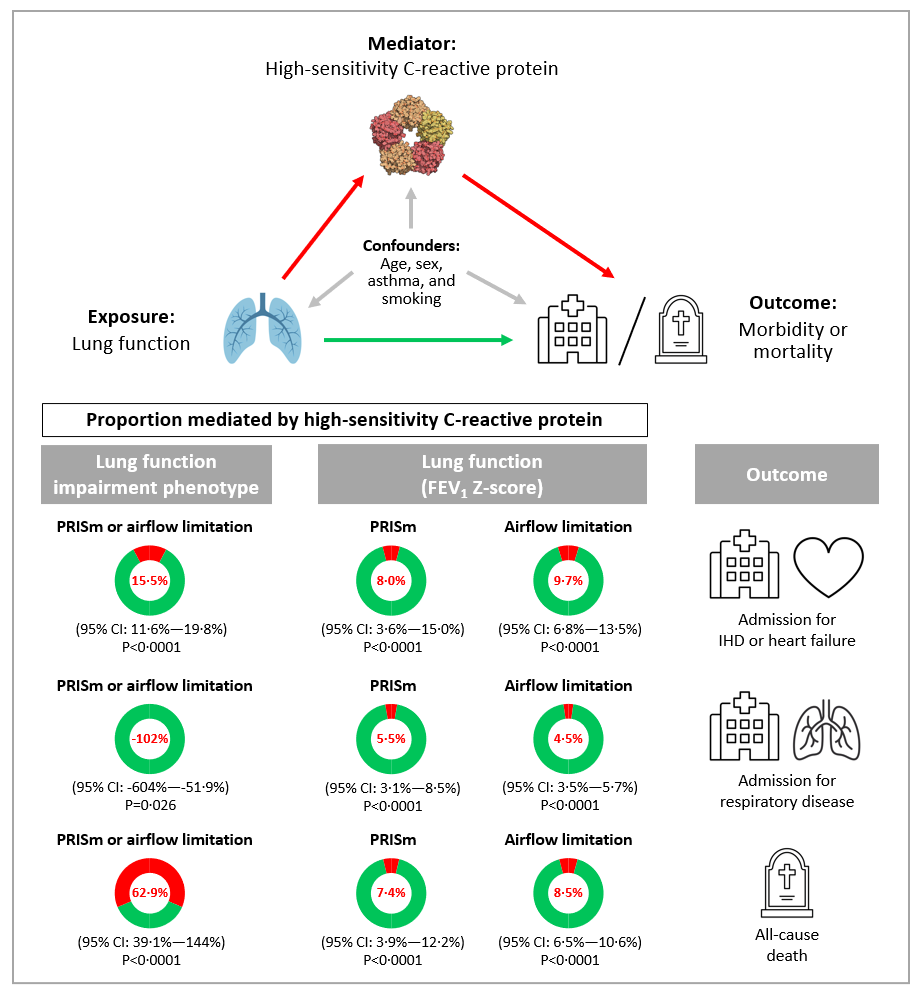


Abbreviations: PRISm, preserved ratio impaired spirometry; IHD, ischemic heart disease; FEV_1_, forced expiratory volume in 1 second.

**Supplementary Figure 10.** Mediation analysis between the exposure lung function (both lung function impairment phenotype and FEV_1_ Z-score), the mediator fibrinogen, and the outcomes ischemic heart disease or heart failure morbidity, respiratory disease morbidity, and all-cause mortality.


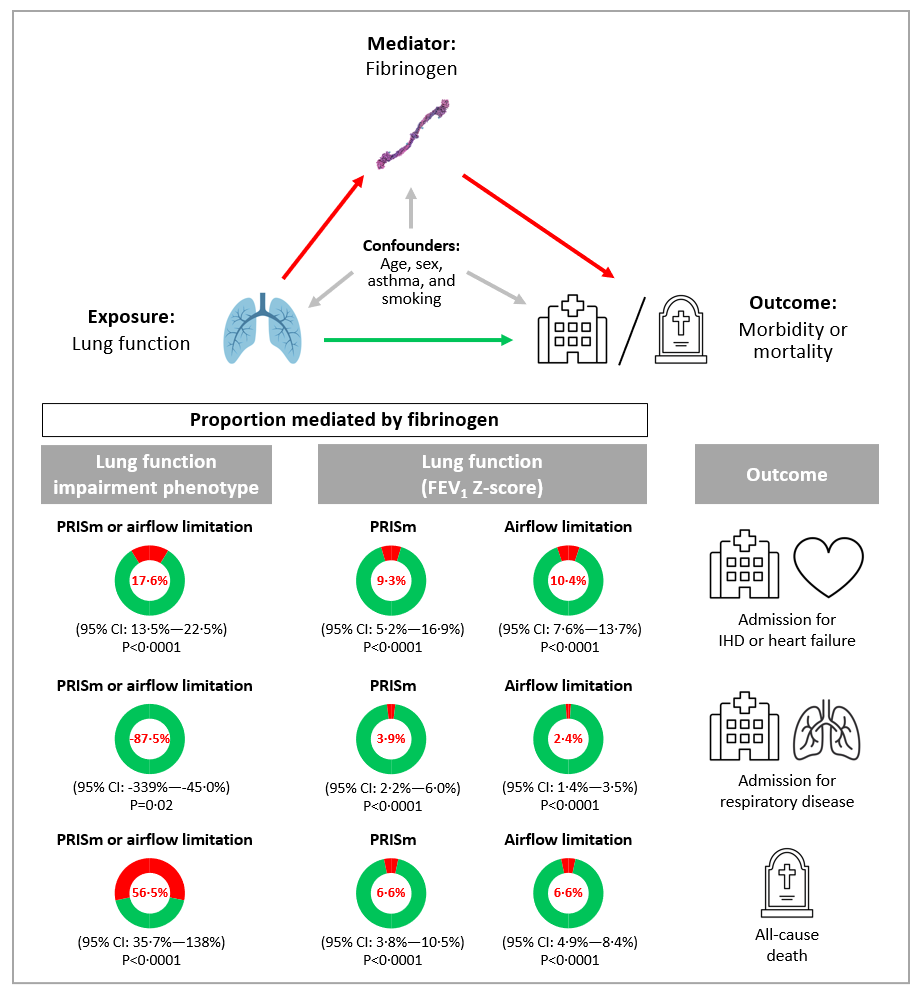


Abbreviations: PRISm, preserved ratio impaired spirometry; IHD, ischemic heart disease; FEV_1_, forced expiratory volume in 1 second.

**Supplementary Figure 11.** Reverse cumulative incidence of ischemic heart disease or heart failure morbidity according to lung function phenotype and the metabolic syndrome.


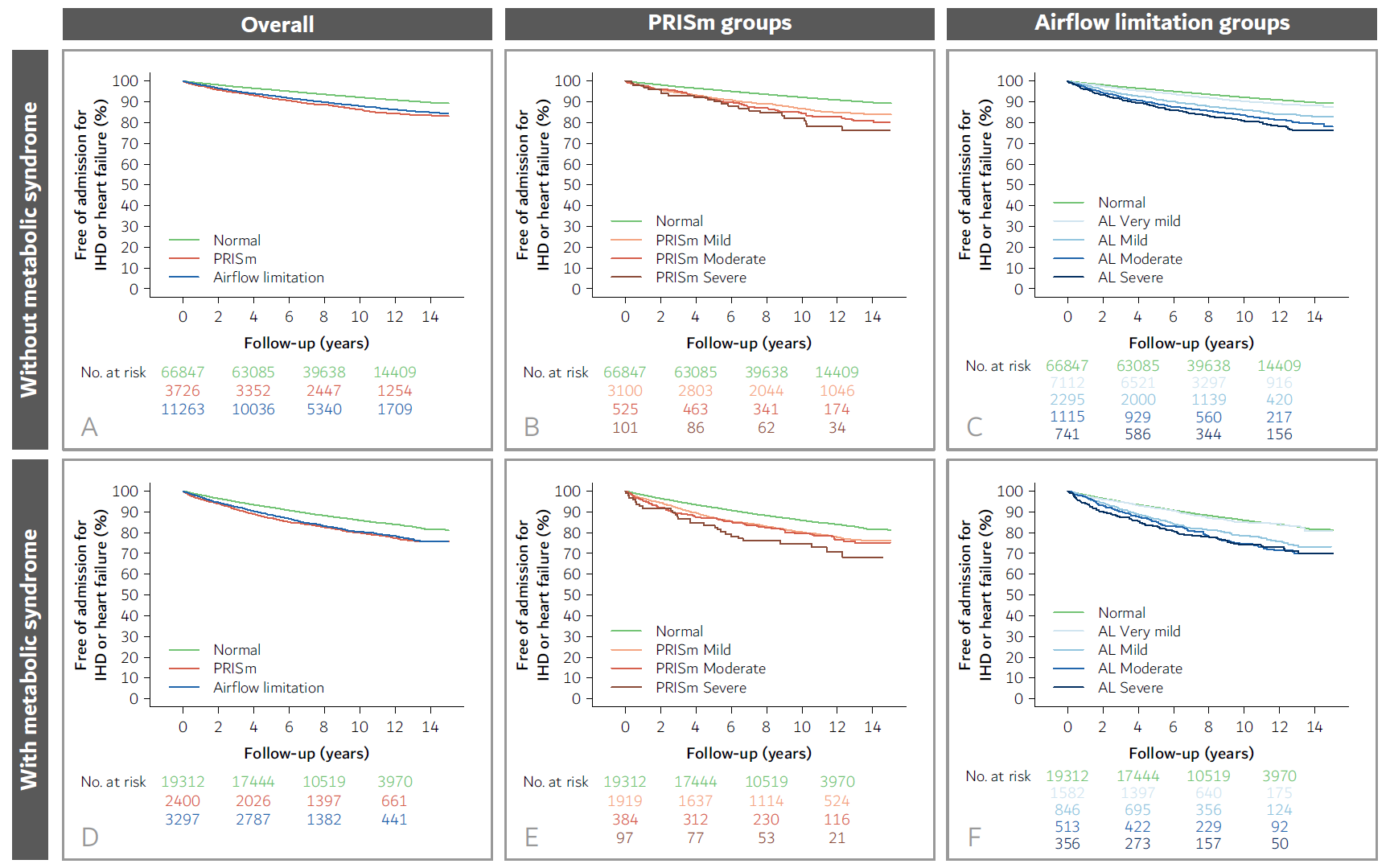


Abbreviations: PRISm, preserved ratio impaired spirometry; AL, airflow limitation; IHD, ischemic heart disease; FEV_1_, forced expiratory volume in 1 second. The severity stages of the lung function impairment were defined according to FEV_1_ Z-scores.

**Supplementary Figure 12.** Reverse cumulative incidence of respiratory disease morbidity according to lung function phenotype and the metabolic syndrome.


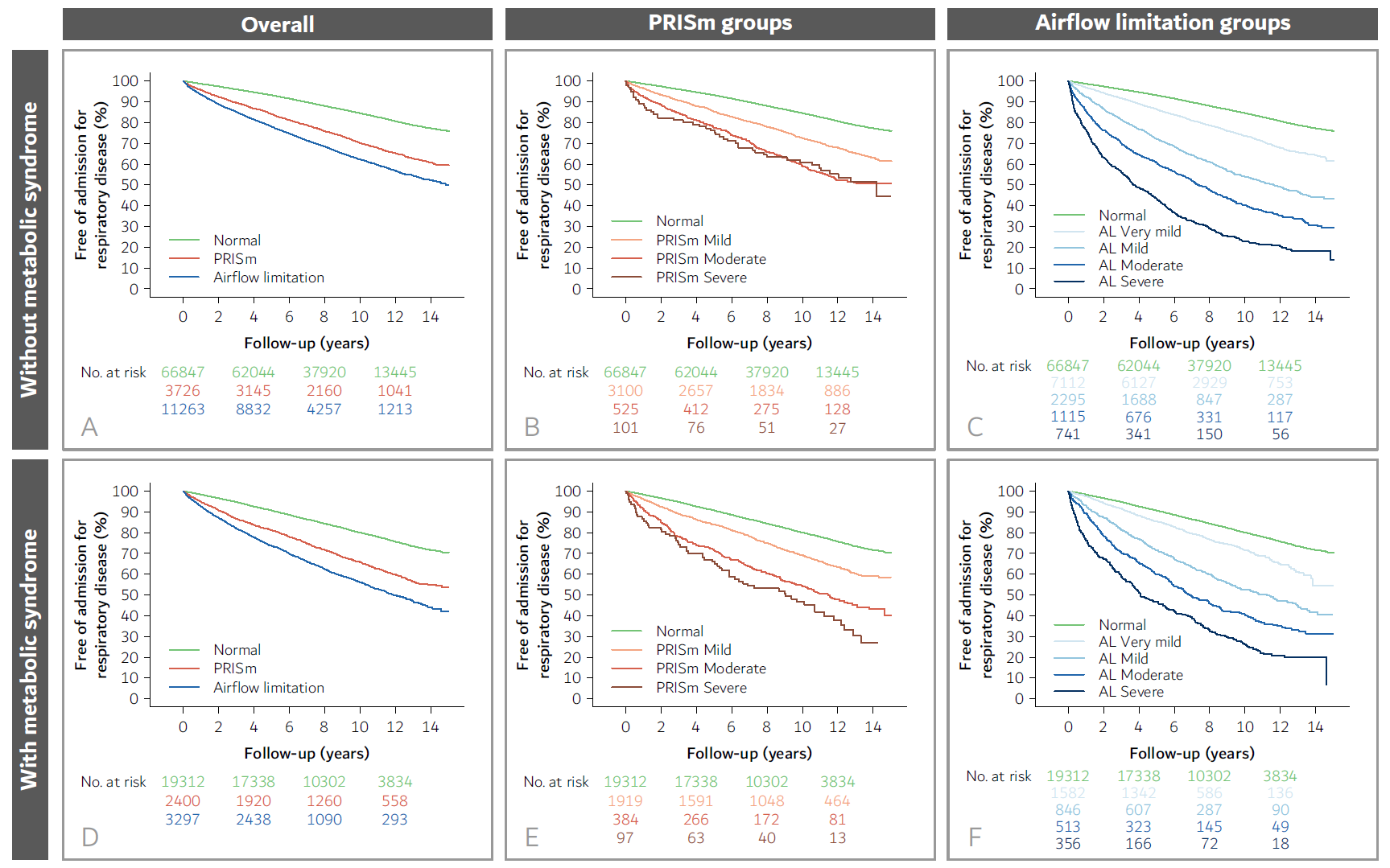


Abbreviations: PRISm, preserved ratio impaired spirometry; AL, airflow limitation; FEV_1_, forced expiratory volume in 1 second. The severity stages of the lung function impairment were defined according to FEV_1_ Z-scores.

**Supplementary Figure 13.** Survival function for all-cause mortality according to lung function phenotype and the metabolic syndrome.


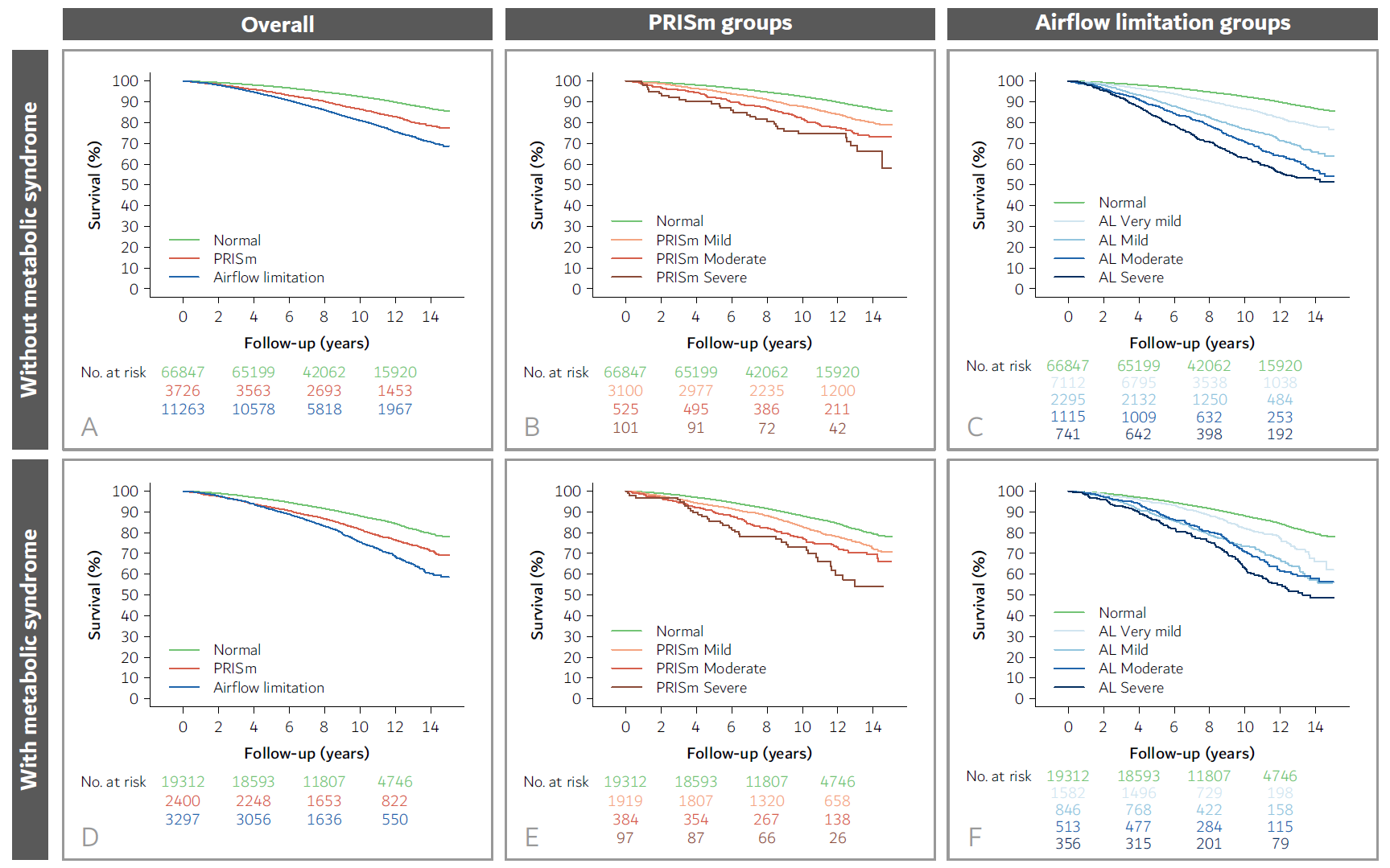


Abbreviations: PRISm, preserved ratio impaired spirometry; AL, airflow limitation; FEV_1_, forced expiratory volume in 1 second. The severity stages of the lung function impairment were defined according to FEV_1_ Z-scores.

**Supplementary Figure 14.** Reverse cumulative incidence of cardiac mortality according to lung function phenotype and the metabolic syndrome.


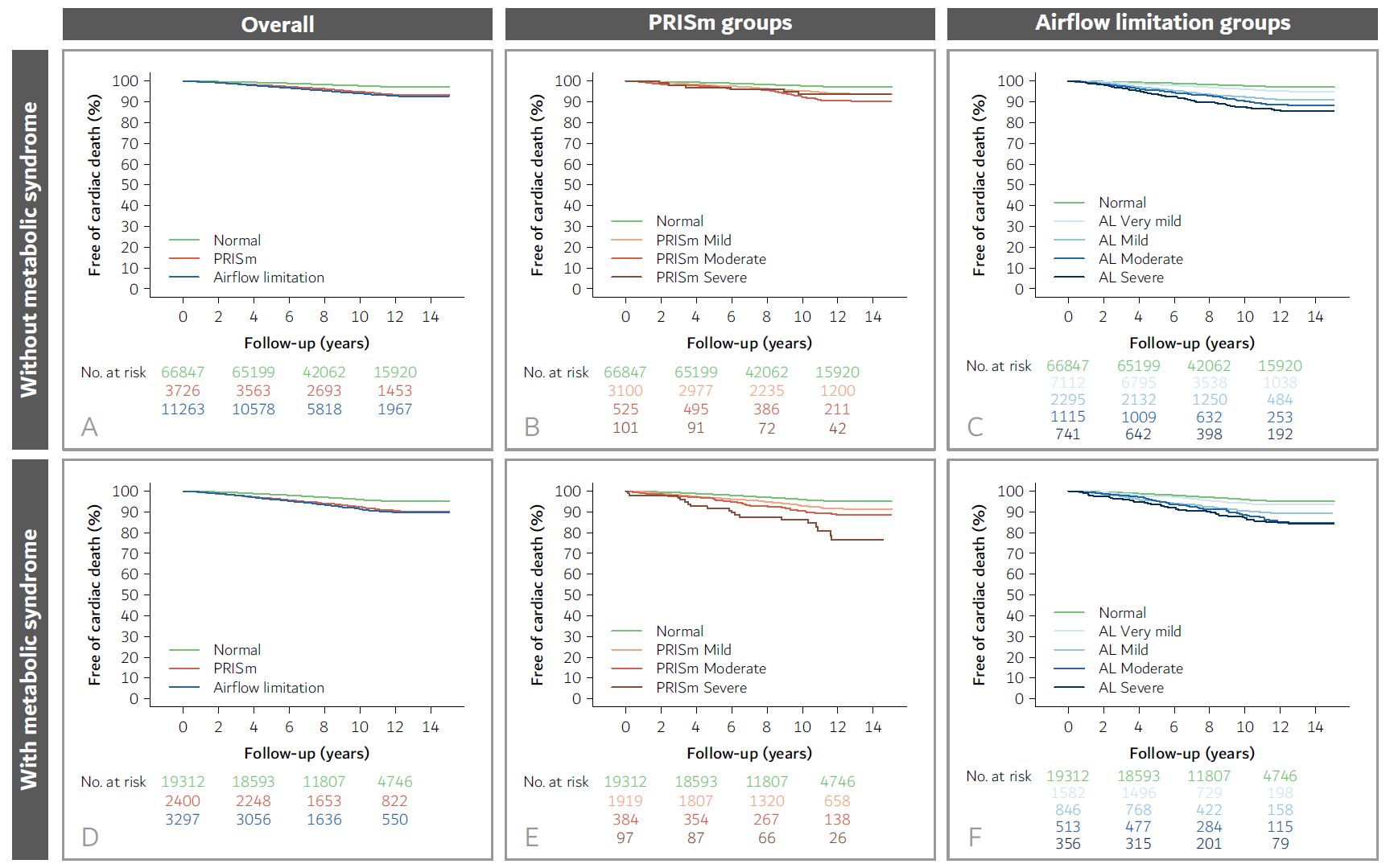


Abbreviations: PRISm, preserved ratio impaired spirometry; AL, airflow limitation; FEV_1_, forced expiratory volume in 1 second. The severity stages of the lung function impairment were defined according to FEV_1_ Z-scores.

**Supplementary Figure 15.** Reverse cumulative incidence of respiratory disease mortality according to lung function phenotype and the metabolic syndrome.


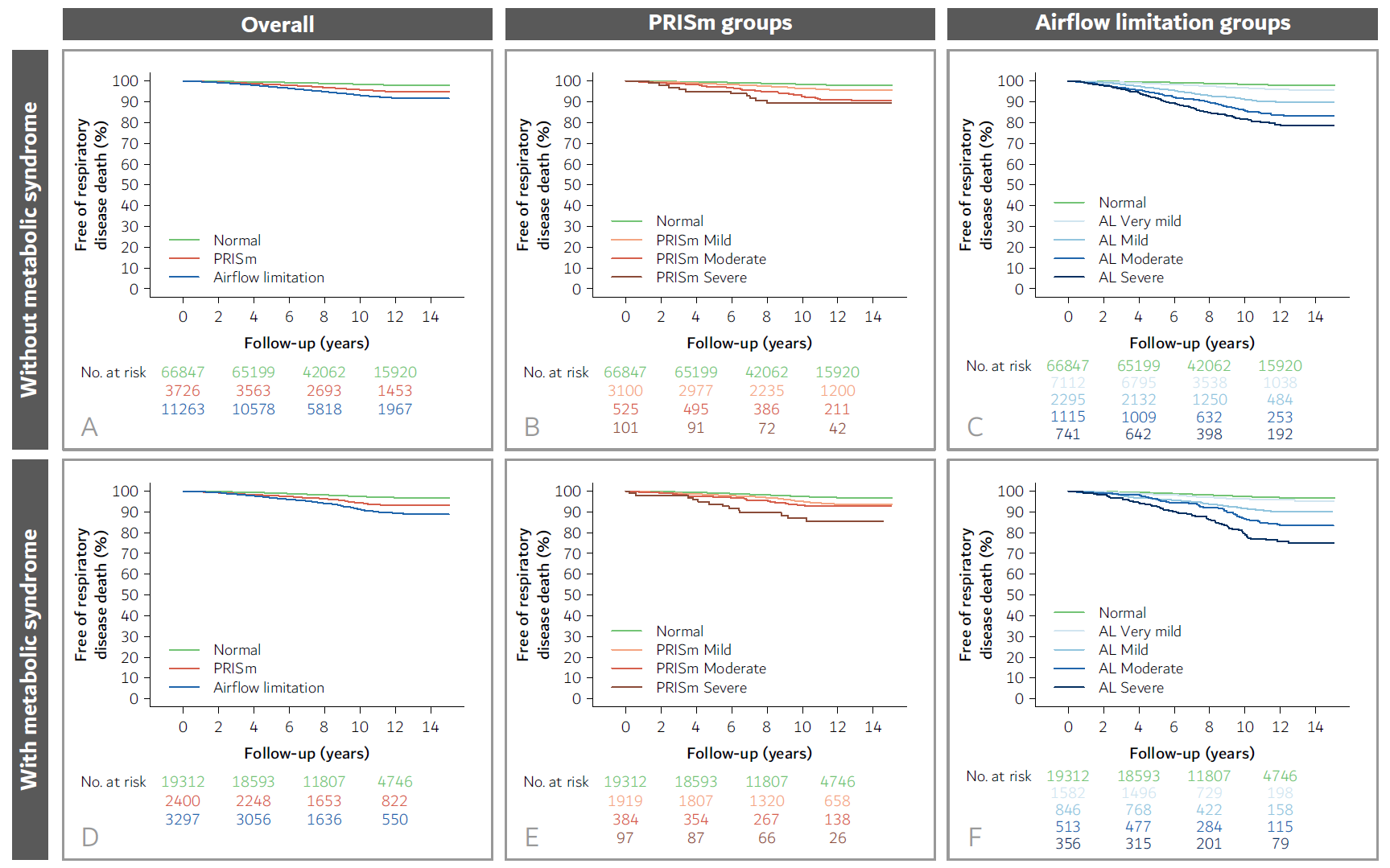


Abbreviations: PRISm, preserved ratio impaired spirometry; AL, airflow limitation; FEV_1_, forced expiratory volume in 1 second. The severity stages of the lung function impairment were defined according to FEV_1_ Z-scores.

**Supplementary Figure 16.** Multiplicative and additive moderation analysis of lung function and the metabolic syndrome in ischemic heart disease or heart failure morbidity, respiratory disease morbidity, and all-cause mortality with adjustment for age, sex, asthma, and smoking.


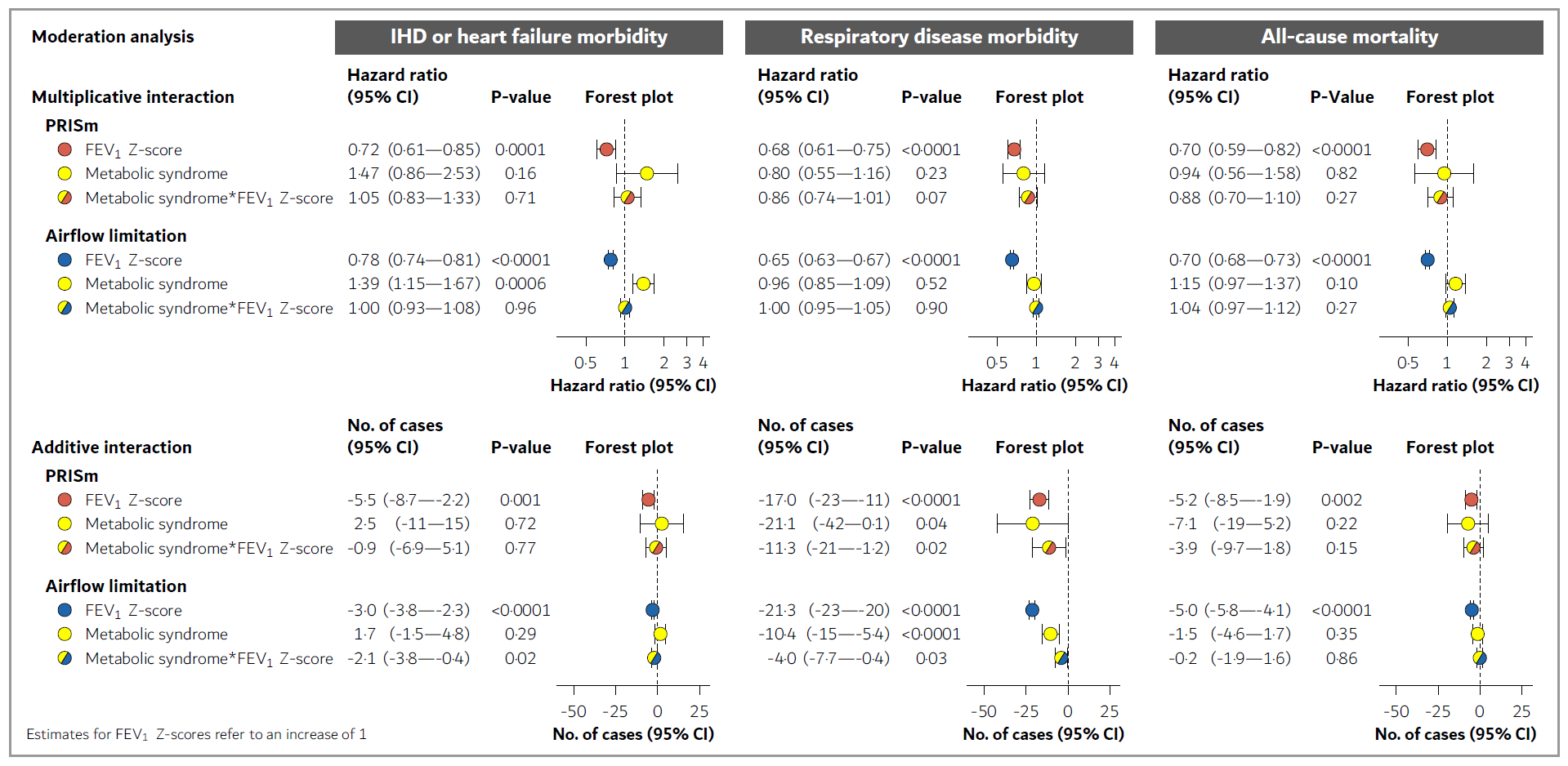


Abbreviations: FEV_1_, forced expiratory volume in 1 second; PRISm, preserved ratio impaired spirometry; IHD, ischemic heart disease; CI, confidence interval. In the additive model, the estimated no. of additional cases is for each outcome presented per 1,000 person years.

**Supplementary Figure 17.** Mediation analysis between the exposures the metabolic syndrome (Panel A), high-sensitivity C-reactive protein (Panel B), and fibrinogen (Panel C), the mediator lung function (FEV_1_ Z-score), and outcomes ischemic heart disease or heart failure morbidity, respiratory disease morbidity, and all-cause mortality.


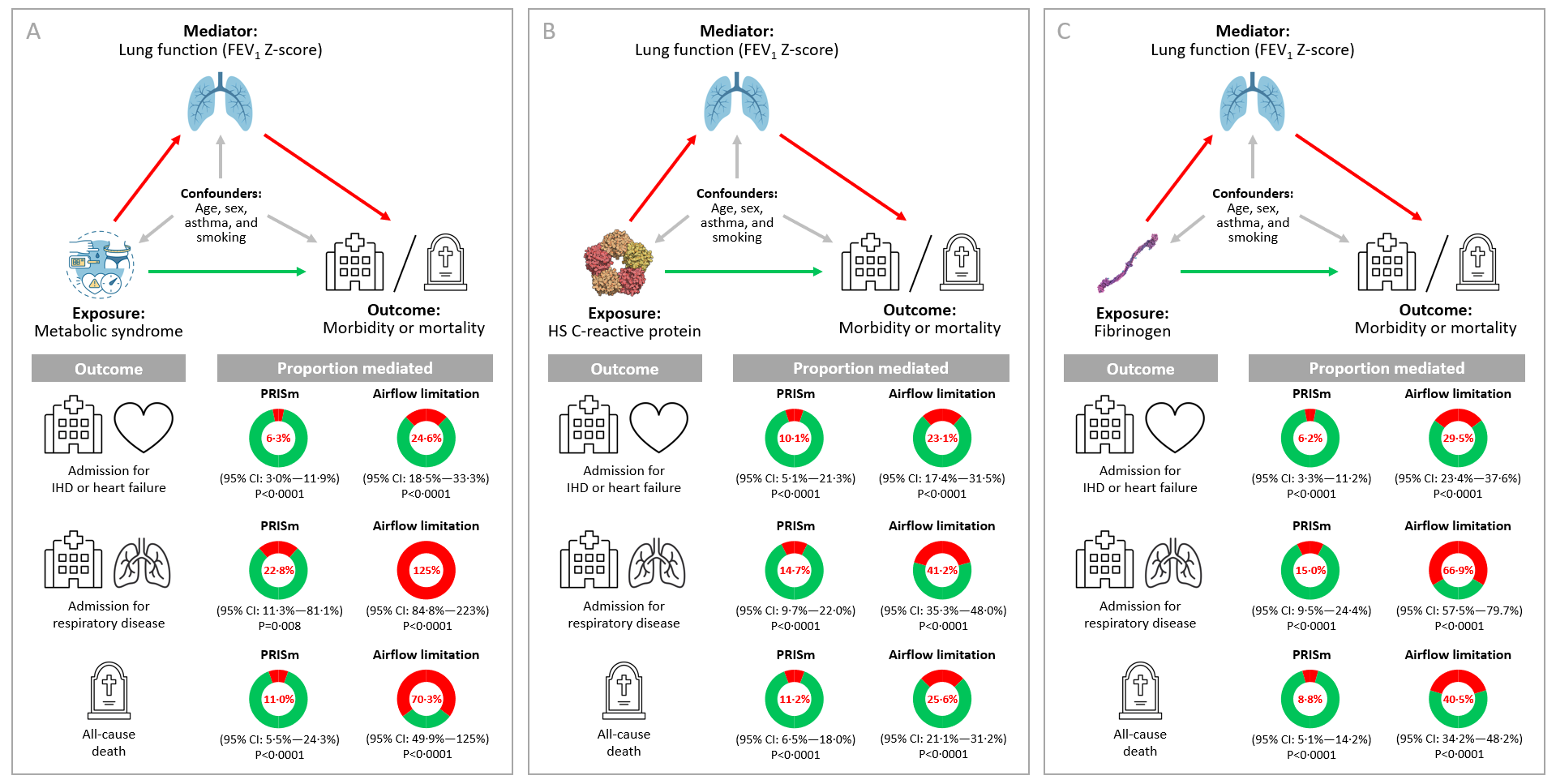


Abbreviations: PRISm, preserved ratio impaired spirometry; IHD, ischemic heart disease; FEV_1_, forced expiratory volume in 1 second.
